# *Quorum sensing* in bacteria: *in silico* protein analysis, ecophysiology, and reconstruction of their evolutionary history

**DOI:** 10.1186/s12864-024-10355-6

**Published:** 2024-05-03

**Authors:** Iñigo de la Fuente, Saioa Manzano-Morales, David Sanz, Alicia Prieto, Jorge Barriuso

**Affiliations:** Centro de Investigaciones Biológicas (CIB Margarita Salas), Department of Microbial and Plant Biotechnology, Consejo Superior de Investigaciones Científicas (CSIC), Ramiro de Maeztu 9, Madrid, 28040 Spain

**Keywords:** *Quorum sensing*, Lux genes, Lifestyle, Ancestral reconstruction

## Abstract

**Background:**

*Quorum sensing* (QS) is a sophisticated cell-to-cell signalling mechanism that allows the coordination of important processes in microbial populations. The AI-1 and AI-2 autoinducer systems are among the best characterized bacterial QS systems at the genetic level.

**Results:**

In this study, we present data derived from *in silico* screening of QS proteins from bacterial genomes available in public databases. Sequence analyses allowed identifying candidate sequences of known QS systems that were used to build phylogenetic trees. Eight categories were established according to the number of genes from the two major QS systems present in each genome, revealing a correlation with specific taxa, lifestyles or metabolic traits. Many species had incomplete QS systems, encoding the receptor protein but not the biosynthesis of the quorum sensing molecule (QSMs). Reconstruction of the evolutionary history of the LuxR family and prediction of the 3D structure of the ancestral protein suggested their monomeric configuration in the absence of the signal molecule and the presence of a cavity for its binding.

**Conclusions:**

Here we correlate the taxonomic affiliation and lifestyle of bacteria from different genera with the QS systems encoded in their genomes. Moreover, we present the first ancestral reconstruction of the LuxR QS receptors, providing further insight in their evolutionary history.

**Supplementary Information:**

The online version contains supplementary material available at 10.1186/s12864-024-10355-6.

## Background

*Quorum sensing* (QS) is a cell-to-cell signalling mechanism dependent on the population density that coordinates the collective behaviour of microbial communities. The process involves the secretion and sensing of small diffusible signal molecules denominated autoinducers (AIs) or quorum sensing molecules (QSMs) [1, 2]. As cell density increases, QSMs accumulate in the environment, triggering coordinated changes in gene expression at the population level after a certain concentration threshold is reached. There are different signal molecules and QS systems, with certain species reacting to a variety of QSMs, while others react to only a limited set. Regulated traits include bioluminescence production, virulence factor secretion, biofilm formation, DNA uptake, sporulation, yeast-to-hyphae transition, or secondary metabolites production.

This phenomenon is well understood in bacteria, where different QS systems have been identified and characterised [3–6]. The best studied (and more common) QS system in Gram-negative species is the so-called autoinducer-1 (AI-1), mediated by *N*-acyl-homoserine lactones (AHLs) as QSMs. This two-component system is composed of a AHL synthase from the LuxI, LuxM or HdtS protein families, and a membrane-bound or cytoplasmic receptor protein from the LuxR, or LuxN families [1, 7, 8]. The receptors act as transcription factors, regulating the response of certain genes. The LuxI-type synthases generate AHLs from *S*-adenosyl-L-methionine (SAM) and an acyl chain, which can be obtained from intermediates of fatty acid biosynthesis (acyl carrier protein, ACP) or from an acyl-Coenzyme A (acyl-CoA) molecule [2, 9]. There are also bacteria that only possess LuxR receptors (denominated orphan or solos), but do not produce the signal molecule. These bacteria are called listeners since they can sense the concentration of AHL in the environment but cannot produce it [10].

Besides the AI-1 system, other QS molecules identified in Gram-negative bacteria [3] are 3-hydroxypalmitic-acid-methylester (3-OH PAME) and (R)-methyl-3-hydroxymyristate ((R)-3-OH MAME) in *Ralstonia solanacearum* [11]; (S)-3-hydroxytriecan-4-one or cholera autoinducer 1 (CAI-1) in some *Vibrio* species [12]; DSF-family autoinducers (derived from *cis*-2-dodecenoic acid) in *Xanthomonas campestris*, *Pseudomonas aeruginosa* and *Burkholderia cenocepacia* [13, 14]; alkyl-hydroxy-quinolones such as 2-heptyl-3-hydroxy-4(1 H)-quinolone (PQS) and 2-heptyl-4-quinolone (HHQ) synthetized by pqsABCDEH cluster in *P. aeruginosa* [15, 16]; dialkylresorcinols (DARs) in *Photorhabdus* spp [17]. ; the pyrazinone class QS molecules including 2,5-diketopiperazines (DKPs) from *P. aeruginosa* [18], 3,5-dimethylpyrazin-2-ol (DPO) from *Vibrio* ssp [6]. . and the recently identified autoinducer-3 (AI-3) in enterohemorrhagic *E. coli* (EHEC) [19].

The second most widespread QS mechanism in bacteria is mediated by a mixture of interconvertible molecules collectively designated as autoinducer-2 (AI-2), which has been described in both Gram-positive and Gram-negative species [2, 20]. The precursor of these molecules is 4,5-dihydroxy-2,3-pentanedione (DPD), synthesized by a protein from the LuxS family. In *Vibrio* ssp. AI-2 needs to be complexed with boron in order to form its active form (2 S,4 S)-2-methyl-2,3,3,4-tetrahydroxytetrahydrofuran-borate (S-THMF-borate). On the contrary, *Salmonella enterica* and *E. coli* AI-2 molecules do not require boron [3]. Furthermore, the soluble periplasmic AI-2 binding protein LuxP is also an essential component of this QS mechanism as it carries out signal transduction inside the cell [21]. Several examples of bacteria that could simultaneously rely on AI-1 and AI-2 QS mechanisms have been characterised [22].

On the other hand, there are QS systems exclusive from Gram-positive bacteria; these are mediated by small autoinducing peptides that are translated in the form of pro-peptides and then exported and processed by extracellular proteases. The active peptides can be internalized by a transporter or detected by a membrane-bound sensor histidine kinase, which initiates a phosphorylation cascade that modulates gene transcription [6, 23]. In the case of *Streptococcus pneumoniae* this system is encoded in the ComACDE cassette [24] while in *Staphylococcus aureus* is in the AgrBDCA cassette [25].

QS mechanisms are not only restricted to bacteria, and have also been identified in fungi [5, 26, 27], where they mediate species-specific responses and enable communication with bacteria [28, 29]. In addition, simultaneously to QS evolution, other microorganisms have developed strategies to inactivate QS systems. These phenomena are called quorum quenching [30], and may have interesting applications such as alternative antibacterial therapies to treat chronic diseases caused by antibiotic-resistant biofilm-forming microorganisms [31, 32].

QS components and their interactions are mainly examined under laboratory conditions; however, their study in different habitats can improve our knowledge on microorganisms’ ecology [33]. Mimicking realistic ecological niches found in nature is difficult, although a few works on QS systems under complex (multi-species bacterial communities) and dynamical conditions (turbulent and laminar flow) have been published [6]. To overcome this hurdle, *in silico* genomic and metagenomic analyses are a good approach to link communication systems with taxonomic diversity and ecological niches [34]. In this sense, the development of high-throughput technologies such as DNA sequencing has generated huge amounts of information that is available in public databases such as NCBI or the Joint Genome Institute (JGI-DOE) [35]. Ecological and evolutionary conclusions can be drawn thanks to the subsequent analysis of these data with bioinformatic tools, such as FastML [36] or PAML [37], expanding our knowledge about microbial physiology and biodiversity [26, 28, 38–40]. These bioinformatics approaches also allow the reconstruction of ancestral sequences based on multiple alignments of known proteins and the statistical inference of extinct sequences [41].

In this work, we adopted a sequential bioinformatic approach to screen for widespread QS proteins from bacterial systems (AI-1, AI-2, AI-3, etc.) in publicly available bacterial genomes. The selected genomes belonged to 92 branches of described bacterial phyla with different lifestyles, which were present in different environments. QS sequences counts were discussed based on microorganisms’ phylogenetic affiliation, habitats and ecological interactions. Finally, molecular evolution of selected QS proteins was inferred, and ancestral sequences reconstructed. Potential properties of the candidates are discussed based on their amino acidic composition and three-dimensional predicted structure.

## Methods

### Genomes database searches

Genomes were retrieved from the server of the Joint Genome Institute, IMG/M (http://img.jgi.doe.gov). Based on the phylogenetic tree built by Hug and collaborators [42], 293 genomes from representative species of the phylogenetic groups and lifestyles ─out of more than 90.000 available bacterial genomes in the database ─ were selected (Table SI). To identify proteins from known bacterial QS systems across different taxa two strategies were used.

First, we implemented an annotation-based approach and searched by term on the IMG/M server by “Gene Product Name”. The search was based on the presence/absence of one of the following terms in gene product names: “Quorum, Sensing, Quorum Sensing, Autoinducer, Homoserine lactone, AHL, signal peptides, AI-1, LptA, AinS, HdtS, AI2, furanosyl borate diester, quinolone, HHQ, AHQ, butyrolactone, LuxI, LasI, YenI, EsaI, RhlI, HdtS, LuxR, LasR, TraR, RhlR, LuxM, LuxLM, AinS, AinR, LuxS, LuxP, pqsD, pqsH, gamma-butyrolactone, ComA, ComC, ComD, ComE, ComR, ComX, PlcR, Papr7, NprR, Rap, SdiA, LuxN, AiiA, LuxM” (Table SII).

The second strategy relied on sequence homology, using as query well characterized reference QS proteins from different families (Table SII), which were compared with putative proteins encoded in the assembled genomes using BLASTp (cut-off value 1e^− 10^). Whenever reference sequences from different bacterial phyla were available BLASTp analysis was performed, using all sequences to maximize the scrutiny. This is the case of LuxP, analysed by using reference sequences from α and γ-proteobacteria species, and the case of pqsH, analysed using sequences from Proteobacteria, Actinomycetes and Firmicutes species (Table SII).

### Candidate sequences analysis

Positive matches from the two screening strategies were selected as candidate sequences and were gathered into separate FASTA files for each protein family. Then, short protein sequences were discarded, and the remaining sequences were scanned for conserved motifs using InterProScan v5 sequence search (Pfam) with default parameters (https://www.ebi.ac.uk/interpro/) [43]. Sequences which did not match conserved motifs from the assigned family were filtered out, and the rest of the candidates were subjected to multiple sequence alignment (MSA) together with reference sequences of each family. MUSCLE algorithm was used for MSA with the following parameters: Gap open − 2.9, Gap extend 0, Hydrophobicity multiplier 1.2, UPGMB, min diag length 24 [44]. Subsequently, a phylogenetic tree was built using the following parameters: Maximum-Likelihood method, JTT model + WAG, uniform rates, all sites, NNI, BioNJ (MEGA11). Sequence clusters were analysed based on their taxonomical affiliations, bacterial lifestyle and habitat.

### Multivariate analysis

The count of positive candidates for the most abundant protein families (Lux) encoded by each genome was transferred to a matrix (Lux positive in Table SI), which was used for multivariate analysis at the server ClustVist (http://biit.cs.ut.ee/clustvis/). The clustering distance was calculated using the Manhattan method with Average linkage method [45]. The results were presented as a heatmap, from which correlations between counts of QS genetic elements, taxonomy and ecological niches could be inferred.

### Reconstruction of ancestral sequences

We selected the most complete phylogenetic trees to infer ancestral candidate sequences. The multiple alignments of LuxR proteins conducted with MUSCLE were manually trimmed to eliminate signal peptides and long gaps using BioEdit 7.1.11. The most suitable evolution model for the set of sequences was double-checked using ProtTest 3.2 [46], and a Maximum-Likelihood tree was built using the WAG (+ F) evolution model allowing 100 bootstrap repetitions (MEGA11). To infer the sequences of the ancestral nodes generated in the previous tree, PAML4.8 software [37] was used with the following parameters: WAG substitution matrix, Empirical + F model, no gaps, amino acids sequence type and verbose detailed. For LuxR family, ancestral nodes 153, 160, 161, 162, 163, 164, 185, 196, 213, 227, 228, 238, 239, 241, and 250 were selected for further analysis. Ancestral sequences from selected nodes were aligned and manually evaluated (Supp. Mat. SI).

### Protein modelling

Predicted sequences for ancestral nodes were manually curated and subjected to three-dimensional modelling by the automated protein homology-modelling server SWISS-MODEL (https://swissmodel.expasy.org/; [47]). The templates used to model the 3D structure of the LuxR proteins were (PDB): SdiA (4Y15), TraR (2Q0O) and QscR (3SZT) from *Escherichia coli*, *Sinorhizobium fredii* and *Pseudomonas aeruginosa* respectively, with QMEAN scores between − 3.42 and 0.29.

Models were comprehensively analysed using PyMol 1.1 (http://pymol.org/), and putative intramolecular tunnels were predicted using Caver analyst 1.0 [48].

## Results and discussion

QS mechanisms are sophisticated physiological adaptations used by microorganisms to thrive in the environment. These systems make communication between individuals from the same or different species possible, enabling a “multicellular” lifestyle [49]. It has been suggested that they could be considered a neo-Darwinian mechanism of evolution, with a primordial role in the development of the first multicellular organisms [50]. Here we have investigated the presence of QS proteins encoded in genomes from most bacterial phyla, and studied their correlations with the habitat lifestyles and phylogenetic affiliations of the microorganisms.

### Screening strategies

In this work, we designed two different strategies to look for QS proteins in a total of 293 genomes representative of each bacterial phylum known to date. As expected, the two search strategies employed rendered unequal results (Table SII). From the ‘Annotation’-based approach, 13,838 hits were identified as putative QS proteins, but only 2880 were considered as candidates after filtering. The main reasons for the great number of false positives initially detected can be attributed to the IMG server pipeline annotation and the use of generic search terms such as “inducer”, “sensing” or “quorum”.

On the other hand, the homology-based strategy consisted in a BLASTp search using 37 reference protein sequences to query for homologue QS gene products with various phylogenetic affiliations. We set up a cut-off value of 1e^− 10^, which produced matches with 20% or higher sequence identity, to account for all possible variability before filtering [26]. This strategy showed a higher proportion of true positives although its main drawback lies in the low availability of reference proteins. Overall, only 879 sequences remained from 19,607 hits after filtering. In this case, certain sequences, such as ComA (AAA69510) and the AHLs receptor YP_204419, generated more than 15,500 false positives in form of histidine kinases, ABC transporters or ATPases.

Combining the filtered results from both strategies and eliminating redundant proteins that presented the same accession number, few matches were found with the LuxM and HdtS families, the quinolone system (PQS 1), the Gram-positive extracellular peptides, and the AI-3 system. In contrast, proteins from the AI-1 (LuxI and LuxR) and AI-2 (LuxS-LuxP) systems were found in 131 of the 293 analysed genomes, while 162 genomes did not match with any of the screened Lux proteins (Table SI). For this reason, we focused on the sequences from AI-1 and AI-2 systems and performed further phylogenetic analyses with the candidates from the families LuxI, LuxR, LuxS and LuxP.

### Phylogenetic analysis

**Fig. 1:**
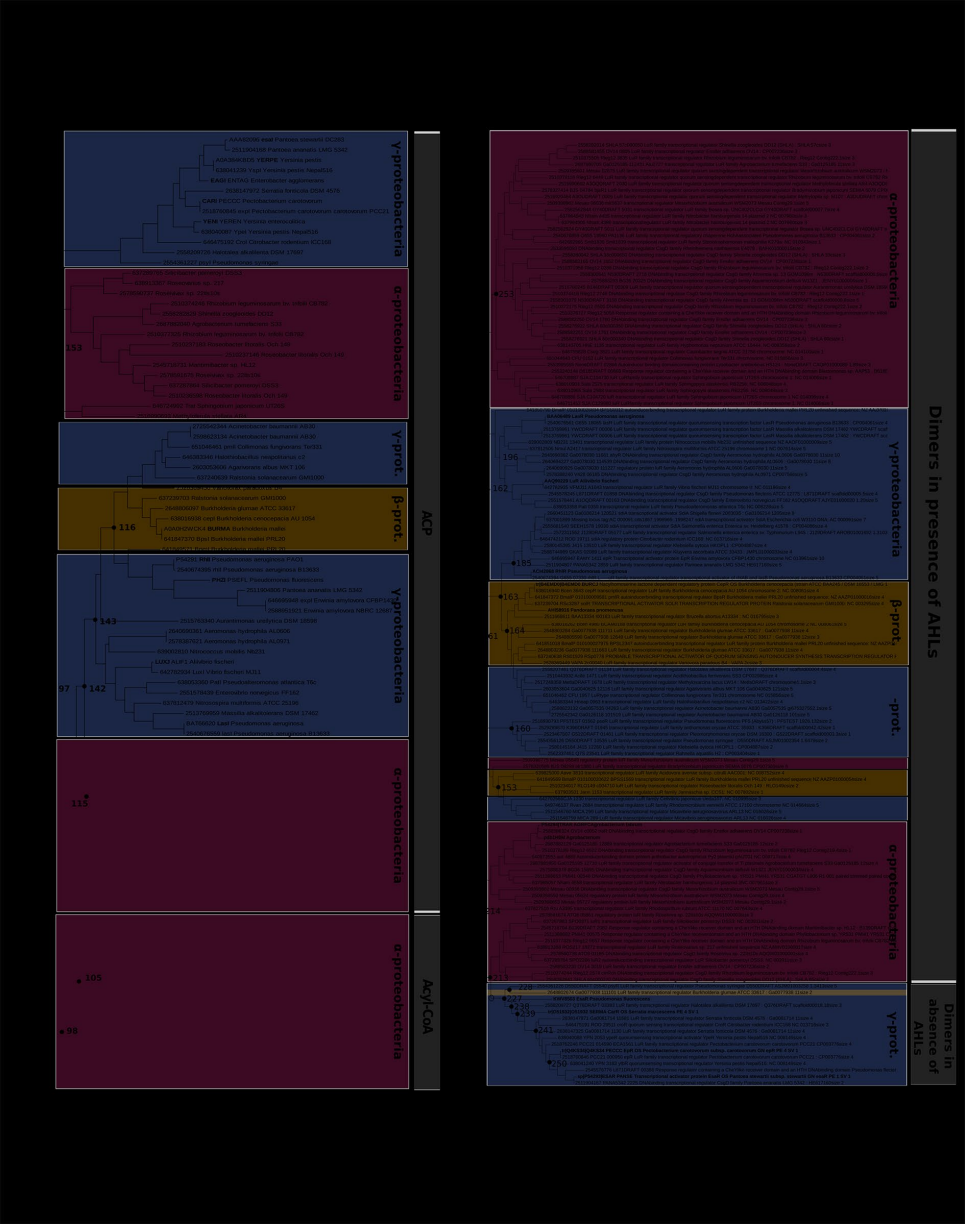
Phylogenetic tree of the proteins from: **A**) LuxI family, **B**) LuxR family. Reference proteins are highlighted in bold. To the right of each tree, shaded in grey, common characteristics of proteins are detailed. Numbered internal nodes were employed for Ancestral State Reconstruction

The phylogenetic trees from the proteins involved in the AI-1 system (LuxI and LuxR families), which use AHLs as signalling molecules, are shown in Fig. 1A and B. All sequences belonged to Gram-negative bacteria from the phylum Proteobacteria [51] despite the fact that LuxI and LuxR genes have been detected in other groups of such as Nitrospira or Bacteroidetes from environmental biofilms [36]. The absence of other bacterial groups beyond Proteobacteria in this analysis may be due to the selected genomes, with little representation of the aforementioned groups Nitrospira (4) and Bacteroidetes (4).

Phylogenetic reconstruction of the LuxI proteins revealed that a set of sequences from α-proteobacteria (Fig. 1A, lower part of the tree) clusters with the reference sequences from *Bradyrhizobium* (Q89VI2) and *Rhodopseudomonas* (Q6NCZ6), forming a monophyletic clade (node 98). These two LuxI synthases are characterized by preferentially synthesizing AHLs from acyl-CoA [9], while the rest of the sequences in the tree group with reference proteins that use ACP as substrate. Therefore, these distinct substrate preferences result in clearly delineated clades. Given that the enzymes that use acyl-CoA are concentrated in specific taxa, whereas those relying on ACP are widespread, it is likely that the capability to use acyl-CoA is a relatively recent trait that emerged after the diversification of Proteobacteria.

Apart from this small clade, the clustering of the ACP-LuxI proteins is primarily determined by the taxonomic origin of the sequence. For instance, the node 115 contains some α-proteobacteria that cluster with the reference sequences of *Sinorhizobium* CCM70616 and *Agrobacterium* TraI, a group of β-proteobacteria comprising sequences from *Burkholderia*, and two sets of γ-proteobacteria, one of which clusters with reference sequences of *Pseudomonas* and *Vibrio* species. In node 153, there is a group of α-proteobacteria related to the order of Rhodobacterales and a cluster of γ-proteobacteria from the order Enterobacterales (*Pantoea*, *Yersinia*, *Enterobacter* and *Pectobacterium*). The apparition of α-proteobacteria and γ-proteobacteria in different branches of the tree may be related to horizontal gene transfer events.

Moreover, there is no direct correlation between the phylogenetic affiliation of the LuxI proteins and the type of AHL they produce, as can be seen in the case of LuxI from *Vibrio* and EsaI from *Pantoea*, which synthesize the same signal molecule (3-oxo-C6-HSL) [52] and grouped into distinct branches of the phylogenetic tree (Fig. 1A).

On the other hand, LuxR family protein members use to have low identity among sequences and in these analyses also proved to be widely represented in the bacterial genomes. Regarding their phylogeny (Fig. 1B), node 213 separates a major group of proteins that need to bind AHLs to become active dimers from a minor pool of proteins that form active dimers in the absence of AHLs (repressors, in the lower part of the tree) [53]. However, there is an exception with the RhlR protein from *Pseudomonas*, which is able to form dimmers both in the presence and in the absence of AHL [54] and clusters in the node 185. This could constitute an evolutionary particularity very interesting to study. The last monophyletic clade (node 227) is taxonomically restricted to γ-protebacteria (except for *Burkholderia glumae*), suggesting the emergence of this type of LuxR receptors after the separation of γ-proteobacteria from the rest of Proteobacteria. It is tempting to speculate that the *B. glumae* copy is the result of Horizontal Gene Transfer from γ to β-proteobacteria.

The sequences of proteins that become active dimers in the presence of AHLs are subdivided into big branches, mostly from α- and γ-proteobacteria. Those from β-proteobacteria are closely clustered and generate small branches of 3–4 sequences. The protein sequence of *Massilla alkalitolerans*, closely related to the reference sequence of *Pseudomonas aeruginosa*, is another exception. In the vicinity of node 163, there is a branch made up of β-proteobacteria, mainly *Burkholderia* sp., which are believed to dimerize in the presence of AHLs.

As mentioned above, the trees from LuxI and LuxR presented in Fig. 1 do not include sequences from phyla Nitrospirae or Bacteroidetes [26]. This fact suggests that not only Proteobacteria [51], but also a diverse array of as-yet-uncultivated bacteria likely possess LuxI/R-type QS systems as shown by other authors [55].

Finally, the phylogenetic tree constructed from the 81 candidate sequences from LuxS proteins (Fig. S1) showed that most of them were Gram-negative bacteria from the γ- and ε-proteobacteria phyla, with a small representation of Bacteroidetes. A few sequences corresponded to Gram-positive species from the phyla Firmicutes and Actinobacteria. Regarding LuxP, there were very few candidate sequences (28) available and all of them clustered as γ-proteobacteria, except for two that grouped as α-proteobacteria (data not shown).

### Taxonomy and eco-physiological groups

The 131 genomes that contained genes encoding QS proteins were classified into 8 groups (Table SI), based on the presence of proteins from the AI-1 (LuxI-LuxR) and/or AI-2 (LuxS-LuxP) systems, and defined as: Group 1, bacteria with proteins from the AI-1 QS system (LuxI and LuxR); Group 2, bacteria with proteins from the AI-2 QS system (LuxS and LuxP); Group 3, bacteria with both, AI-1 and AI-2 QS systems; Group 4, bacteria with only LuxR receptors or with more LuxR proteins than LuxI synthases (AI-1 listeners); Group 5, bacteria with only LuxP receptors or with more LuxP proteins than LuxS synthases (AI-2 listeners); Group 6, bacteria with AI-2 QS system and LuxR receptors (AI-2 + AI-1 listeners); Group 7, bacteria with AI-1 QS system and LuxP receptors (AI-1 + AI-2 listeners); Group 8, bacteria with higher amount of LuxR and LuxP proteins than LuxI and LuxS synthases (AI-1 and AI-2 listeners).

**Fig. 2:**
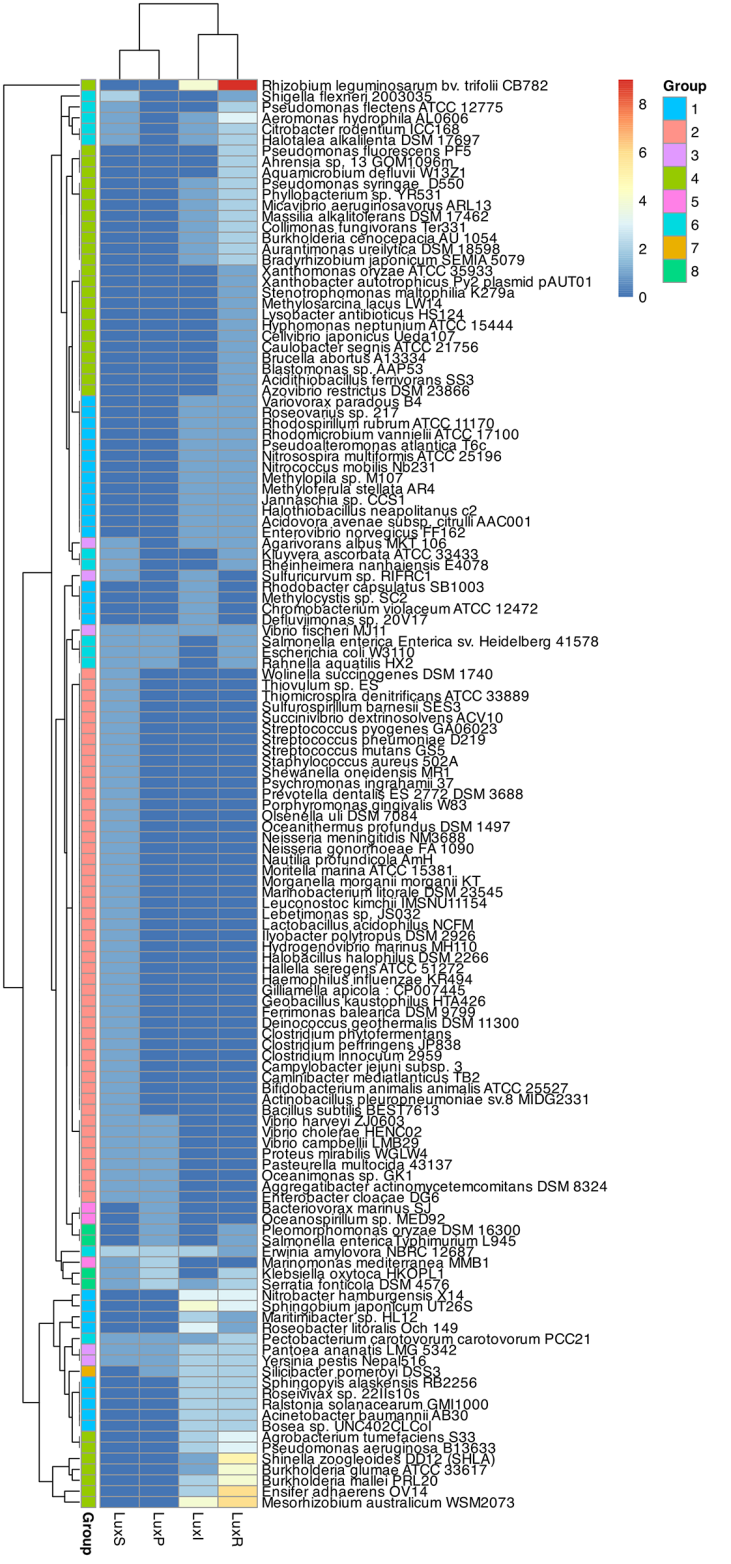
Clustering analysis and heatmap representation of the QS protein candidates found in the 131 genomes. Groups 1 to 8 gather species with different profiles of proteins from the AI-1 and/or AI-2 QS systems

Using the matrix representing the abundance of each Lux protein in each genome (Table SI), we performed a clustering analysis and constructed a heatmap (Fig. 2), where the Y axis represents proteins from AI-1 and AI-2 QS systems, and the bacterial genomes are clustered in the X axis according to the presence/absence pattern of these proteins.

Further analysis of the clustering results revealed that most of the microorganisms included in the different categories shared lifestyles or metabolic traits. Group 1 (AI-1 QS system) includes 26 species belonging to the phylum Proteobacteria (62% α-, 19% β- and 19% γ-proteobacteria). The bacteria in group 1 share two common traits: (i) they participate in the nitrogen cycle (N_2_-fixers or denitrifying bacteria), and (ii) they can live in contaminated habitats (seawater or soil) and are involved in the bioremediation of pollutants.

Group 2 (AI-2 QS system) includes Gram-positive (Actinobacteria and Firmicutes) and Gram-negative (Bacteroidetes, β, ε, or γ-proteobacteria and fusobacteria) anaerobic bacteria whose lifestyle is usually linked to a host (pathogens), as well as some extremophiles (*Deinococcus*-*Thermus*). When performing the multivariate analyses without the LuxP protein sequences the clustering was not affected due to the low number of these sequences.

Group 3 (AI-1 + AI-2) only includes 5 bacterial species (4 γ- and 1 ε -proteobacteria) from different habitats, but mostly associated with a host.

Group 4 (AI-1 listeners) includes 30 species of Proteobacteria (53% α-, 20% β- and 27% γ-proteobacteria) that seem to have a high impact on the N_2_ cycle, which correlates them with bacteria from group 1. Most of them can fix N_2_ or reduce nitrate, and the main difference between both categories refers to the lifestyles of their members. With the exception of a few species that have an aquatic, free lifestyle, bacteria from group 4 are host-dependent and live in the rhizosphere (as plant symbionts or pathogens) or are human pathogens. Given that the species categorized in both groups share the same environments, the presence of higher levels of LuxR than of LuxI proteins in group 4 might be a determinant of their lifestyle (host-dependent vs. free in group 1).

Groups 5 (AI-2 listeners) and 7 (AI-1 + AI-2 listeners) comprise free living marine species and are very small, with 3 representatives in the first group (*Marinomonas mediterranea* MMB1, *Oceanospirillum* sp. MED92 and *Bacteriovorax marinus* SJ) and only one in the second (*Silicibacter pomeroyi* DSS3). These data suggest that, by sharing the same environment, both groups are more related to each other than either of them are to group 2 (AI-2 speakers, with anaerobic host-dependent lifestyle).

Group 6 (AI-2 + AI-1 listeners) and group 8 (AI-1 listeners + AI-2 listeners) cluster γ-proteobacteria, 11 and 3 respectively (except *Pleomorphomonas oryzae* DSM 16,300 belonging to group 8), whose lifestyle is mainly pathogenic (plant, animal or human).

The correlation environment-QS system deduced from these data suggests that the atmosphere in which bacterial communities live has a strong influence on the development of a specific signaling system. Many species produce incomplete QS systems and synthesize only a receptor. This is particularly notable in the case of the AI-1, which has large number of listener species that cannot produce the AHL. The presence of defective QS systems associated with certain habitats suggests that the proper functioning of these systems is not essential for the survival of bacteria in these environments [56].

### LuxR ancestral sequences reconstruction

**Fig. 3:**
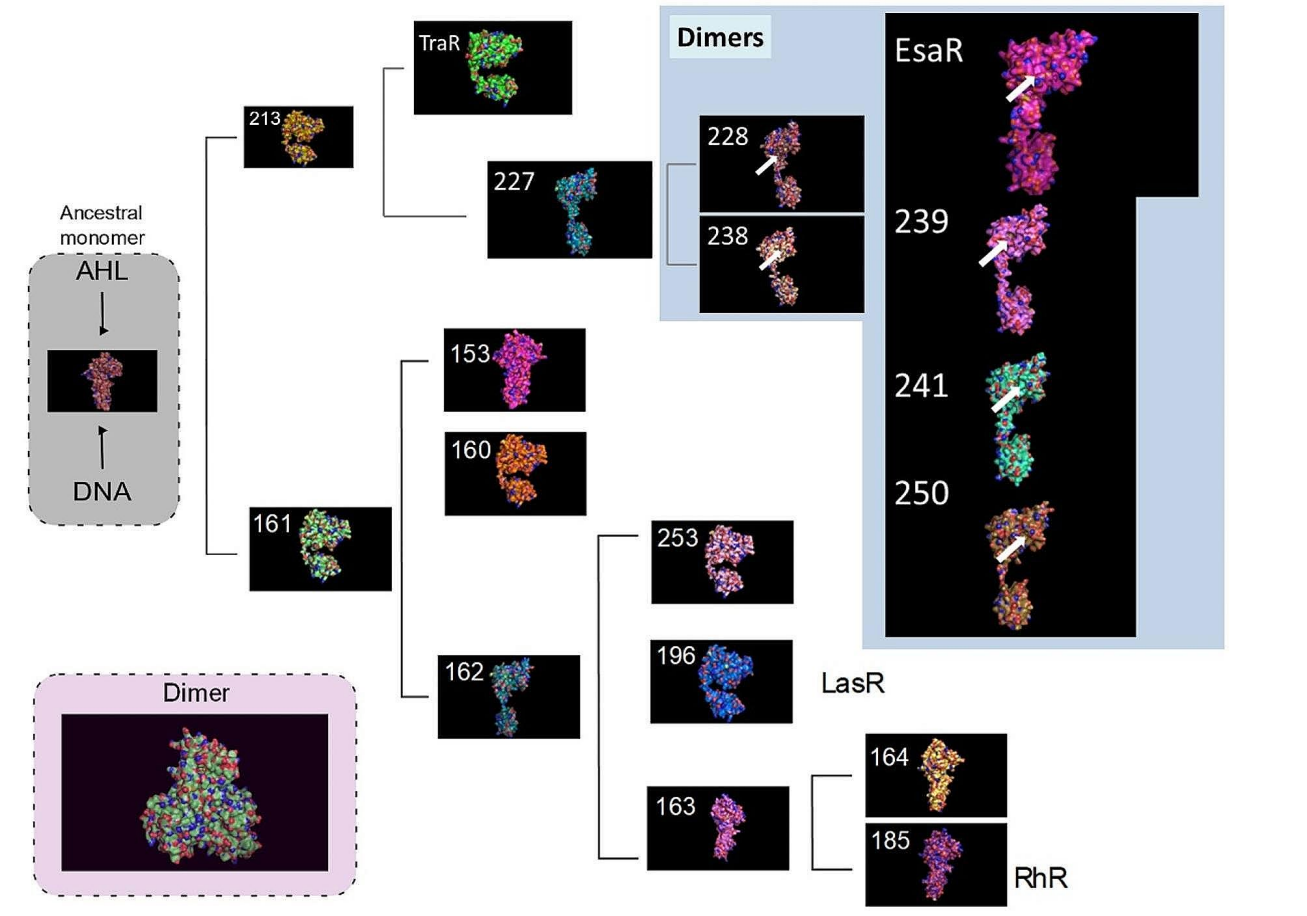
Three-dimensional models of putative ancestral proteins from the LuxR family, with the surface representation of the cavities and tunnels. White arrows point to the cavity to which AHL is suspected to bind in the dimeric inactive form of this set of proteins

To understand the evolutionary history of the most abundant proteins derived from the searches, we built an evolutionary tree of the LuxR proteins family and inferred ancestral sequences. The most representative nodes were selected for 3D modelling of the proteins. Figure 3 depicts the 3D models and their representative features. It can be seen that the most ancestral proteins are monomeric in the absence of AHL and are activated by acquiring a dimeric form in the presence of the autoinducer, thus being able to bind DNA and act as transcriptional activators. The appearance within the group of γ-proteobacteria of LuxR proteins that remain as monomers in the presence of AHL but dimerize and act as repressors in its absence seems to be a more recent evolutionary event (starting from node 227 in Fig. 1).

The interchange between the inactive monomeric and the active dimeric form takes place after the binding of the signal molecule. Looking at the models, it is interesting to note that the pool of LuxR proteins that are monomeric in the absence of AHL displays a cavity where the signal molecule may bind, while this cavity is not accessible anymore in the dimer. On the other hand, models of LuxR proteins that are active dimers in the absence of AHL (from node 227 onwards) show an accessible cavity in the dimeric form (Fig. 3). In this case, the binding of an AHL molecule would generate the inactive form, incapable of binding to DNA [53].

## Conclusions

In this study, we conducted a comprehensive analysis of bacterial AI-1 and AI-2 QS systems, comparing them across various taxonomic groups, habitats, and lifestyles. Phylogenetic analysis of LuxI proteins revealed two well-defined monophyletic clades that grouped proteins that synthetize AHLs from acyl-CoA and ACP, respectively. The former was taxonomically restricted to α-proteobacteria, suggesting its emergence after Proteobacteria diversification. A similar pattern was observed for LuxR, with one clade grouping proteins requiring AHLs for dimerization and a second, mostly restricted to γ-proteobacteria, that was dimeric independently of the QSM. The clustering in these trees suggests horizontal gene transfer between lineages. We also examined the distribution of QS proteins among ecophysiological groups, revealing correlations between habitat/lifestyle and prevalent QS systems.

Furthermore, we reconstructed ancestral LuxR sequences and performed 3D protein modeling on selected nodes. Our findings suggest that the ancestral LuxR was an inactive monomer in the absence of AHL, with an exposed cavity for binding of the autoinducer that would be concealed after attachment of AHL and dimerization.

These results pave the way for wet-lab experiments to increase our understanding of communication processes in bacterial biofilms, which will be useful in the development of new antimicrobial therapies. Notably, phylogenetic conflicts (incongruences between the gene and bacterial species trees) among QS genes raise questions about the role of horizontal gene transfer in QS evolution across lineages. Moreover, dating phylogenetic trees can help determine the relative emergence of each QS system.

To our knowledge, this is the first report on the reconstruction of LuxR ancestors. We believe that this research can enhance our understanding of interactions within microbial communities in different ecological niches, and contribute to the development of potential antimicrobial therapies.

### Electronic supplementary material

Below is the link to the electronic supplementary material.

Supplementary Material 1

Supplementary Material 2

Supplementary Material 3

Supplementary Material 4

## Data Availability

The datasets analysed during the current study are available in the Joint Genome Institute (IMG/M) repository (http://img.jgi.doe.gov).
