# Supplementary figures and images for: Quorum sensing in bacteria: in silico protein analysis, ecophysiology, and reconstruction of their evolutionary history
Source: BMC Genomics. 2024 May 3;25:441. doi: 10.1186/s12864-024-10355-6 (PMC11069264; doi:10.1186/s12864-024-10355-6)

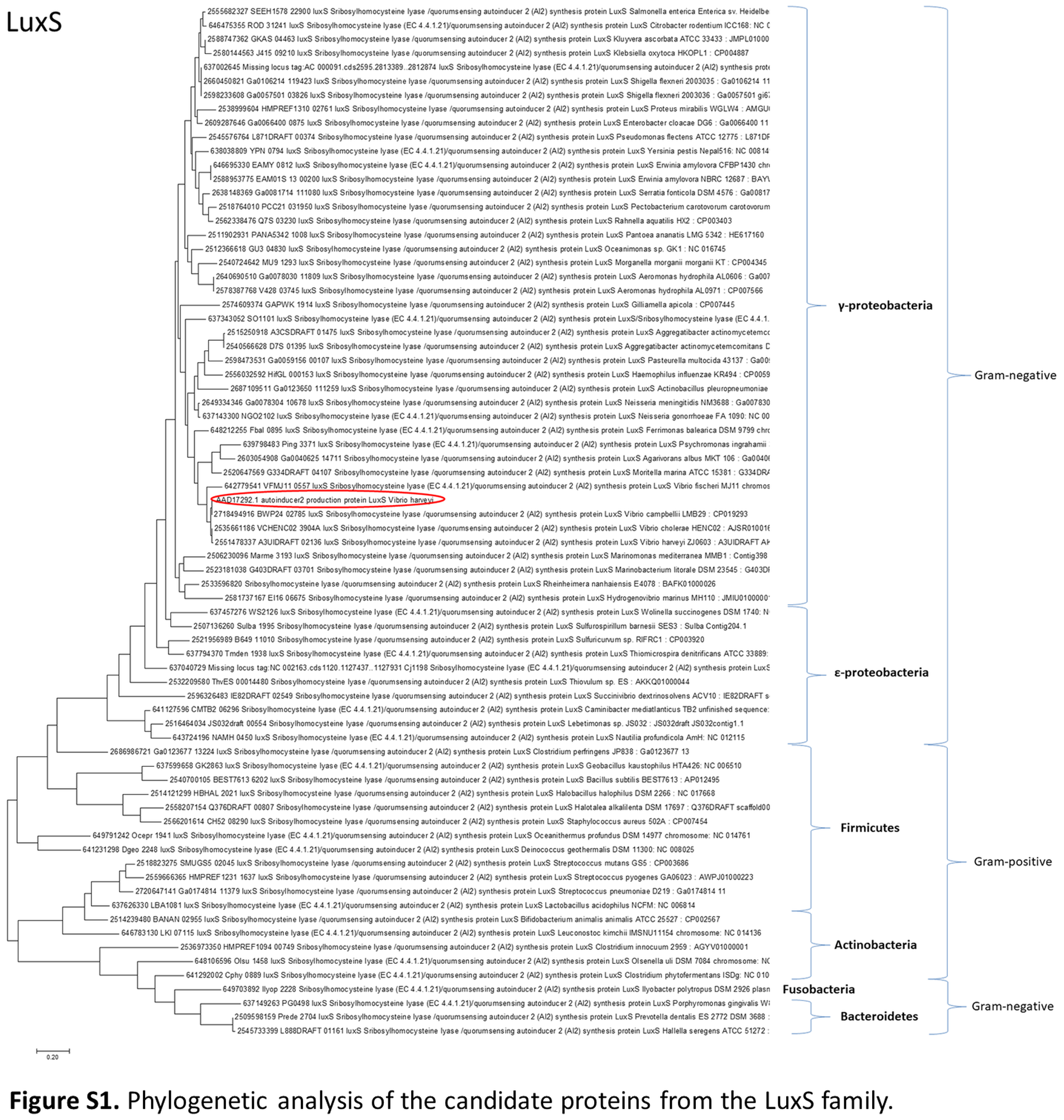

Supplement: Supplementary file 1 — Supplementary Material 1 [file 12864_2024_10355_MOESM1_ESM.png]
