# Supplementary material for: Quorum sensing in bacteria: in silico protein analysis, ecophysiology, and reconstruction of their evolutionary history
Source: BMC Genomics. 2024 May 3;25:441. doi: 10.1186/s12864-024-10355-6 (PMC11069264; doi:10.1186/s12864-024-10355-6)
Supplement: Supplementary file 4 — Supplementary Material 4 [file 12864_2024_10355_MOESM4_ESM.docx]

**Supp. Mat. SI.** Ancestral sequences from the LuxR family.

**LuxRAncestralNodesTrimmed**

>node152

LARSAPELFRLLARFARVFGLDLFAYGRGRLPPTVPGLGLLEGEYPREWLREFLEGELYLACPVFRHAFRLLSPFLWRYPDVSLKLVPQERPLLEELEAAGLRLGLTEPLHGPGGRLDAVCLLPPGHPPYLAVAPPDDLAGANLLALSFHDRYYRRLTEHGVILEPRERACLCWAGRGITVWEPLTALGLVVRRFSFALPFAVRLLRPLHRPLAALVAAFLGLL

>node153

QARSAEELFELFAKAARQFGFDHFAYGATDLPSSKPGSGVLSGNYPDEWVEHYLERNYYLIDPVHRHAYRASSPFRWSDVQSQMKLEKQQRRIMEEAEEAGLRNGLTVPLHGPGGRIAAVSLAASSHAPYSAEAPPDALAKAQLLAIQFHERYYRRLQNQGVKLTPRERECLTWAARGKTSWEIAQILGISEHTVNFHLKNAMRKLNATNRTLAAVKAIRLGLI

>node154

QAKSAEELFSLFAKAASQFGFDRFVYGMTDHPSLKKGHGILSGNYPDDWVEHYFERNYYHIDPVHRHAYRASSPFTWSDLKSQMKLSKQQRRIMHEAEEAGLRNGLAVPLHGPGGQIAAVGLAASSHAPYSAEAPPDALDKAQLLAIQFHVAYYRRLENQGVHLTPREREVLTWAAAGKTDSEIAQILGISEHTVDFHMKNVFKKLNANNRTLAAVKAIRLGLI

>node155

QAKSAEELFSLFAKAASQFGFDRFVYGMTDHPSLGKGHGIVCGNYPDDWVEHYFERNYYHIDPVHRHGYRASSPFTWSDLKSQMKLSKQQRRIMHEAEEAGLRNGLAVPLHGLNGQIAAVGLAASSHAPYSAEAPPDALDKVQALAIQFHVAYYRRLENQGVHLTPREREVLTWAAAGKTDSEIAQILGISEHTVDFHMKNVFKKLNANNRTLAAVKAIRLGLI

>node156

RAKSADDLFSILAKAASKHGFDRCVFGMTDHPDIGKGFGIVCGSYPDDWVQHYFERNYRHIDPVAMHGSRRSEPFLWSDLKDLMKLSKQQRALMFLAEEAGLRNGLAVPLHGLNGQIAAVGLAASSHAPYSSEEPPYDLDMVAAYANHFYVAYYKRLENQGVHLTPKEREVLTWAAAGKTDSEIASILNMSRNTVDAHMRKVFKKLNANSRVLAAVKAISLGLI

>node157

QARSAEELFELFAKAARQFGFDHFAYGATDLPSSKPSSGVLSGNYPDEWVEHYLERNYYLIDPVHRHAYRASSPFRWSDVQSQMKLEKQQRRIMEEAEEAGLRNGLTVPLHGPGGRIAAVSLAASSHAPYSAEAPPDALAKAQLLAIQFHERYYRRLQNQGVKLTPRERECLTWVARGKTSWEIAQILGISEHTVNFHLKNAMRKLNATNRTLAAVKAIRLGLI

>node158

QARSAEELFELLAKAARQFGFDHFAYGAVDLPSSKPSSLVLSGNYPDEWVERYLERNYYLIDPVHRHCHRSSSPFRWSDVQSQMKLEKQQRRIMEEAREAGLRNGLTVPLHGPGGRIAAVSFAASSHAPYSAEAPPDALAEAQLLAIQFHERVYRRLQNQGVKLTPRERECLQWVARGKTSWEIAQILGISEHTVNFHLKNAMRKLGATNRTQAVVKAIRLGLI

>node159

QARSAEELFELLAKLARQFGFDHFAYGAVDLPSSKPSSLVLSGNYPDEWVERYLEQGYYKIDPVVRHCRRSTSPFRWSDVQSQQKLEKQQRRVMEEAREFGLRNGLTVPLHGPNGRFAAVSFAASSHAPYSAEAPPDAMAELQLLAIQFHERVYRRLQSQGVKLTPRERECLQWVAEGKTSWEIAQILGISEHTVNFHLKNAMRKLGATNRTQAVAKAIRLGLI

>node160

QASSAEELFELLAKLARQFGFDHFAYGAVPLPSSKPSSLVLSGNYPDEWVERYLEQGYYKIDPVVRHCRRSTSPFVWSDEQSQQKLEKQQRRVMEEAREFGLRNGLTVPIHGPNGRFAALSFCASDHPPYSAEAPPEAMAELQLLAIYFHERVYRRLQSQGVKLTPRERECLQWAAEGKTSWEIAQILGISERTVNFHLKNAMRKLGATNRTQAVAKAIRLGLI

>node161

QASSAEELFELLAKLARQFGFDHFAYGAVPLSSSKPSSFVLSGNYPDEWVERYLEQGYYKIDPVVRHCRRSTLPFVWSDEQSQSKLEKQQRRVMEEAREFGLRNGLTVPIHGPNGRFAMLSFCASDRPPYSAEAPPEAMAELQLLAIYFHERVYRRLQSQGVKLTPRERECLQWAAEGKTSWEIAQILGISERTVNFHLKNAMRKLGATNRTQAVAKAISLGLI

>node162

QASSEEELFELLAKLARQLGFDHFAYGKVPLSLSKPSVFILSGNYPEEWMERYDEQGYYKIDPVVQHCMRSTLPLVWSDELFQSKREKQQRRVMEEAREFGLRNGLTVPIHGPNGRFGMLSFCASDRPEYSAEAPPEAMAELQLLANYLHEAVYQRLQPQGVKLTARERECLQWAAEGKTSWEIAQILGISERTVNFHLKNAMRKLGATNRTQAVAKAISLGLI

>node163

AASSEEQLFELLAKLARRLGFDYFAYGRVPLPLSKPKVFILSGNYPEAWMERYQEQGYYEIDPVVQHGMRSQMPVVWSDELFSSARETEDRRLWEEARDFGLRHGVTQSIRAPNGAFGMLSFCARDRPRYSISAPPEIELQLQLLANLLHAALYSRLMPEGVKLTAREREVLQWTAEGKTSGEIAQILSISERTVNFHLKNVMKKLGATNKTQAVAKAAALGLI

>node164

AAESEEQLFEQIAAVARRLGFEYCAYGRVPLPLSKPKVVILSGNYPEAWMERYQEQGYLEIDPTVRHGMRSQMPVVWSDELFSSAPETEDRRLWEEARDFGLRHGVAQSSRAANGAFGMLTFCARSGPRYPISAPPEIELQLQWLANLAHAALYSRLMPEGVKLTAREREVLRWTAEGKTAGEIAQILSISERTVNFHVKNVMKKLGATNKTQAVAKAAALGLI

>node165

SAESEEQLFEQIAAVARALGFEYCAYGRVPLPLSKPKVVILSGNYPEAWQQRYQEQGYLE

IDPTVRHGMRSQMPVVWSDELFSSAPETEDRRFWEEARSFGLRHGWAQSSRDANGTFGML

TLCARSGERYPISAPPEIEYQLQWLANVAHAALYSRLMPEGVKLTSREREVLRWTADGKT

AGEIAQILSISERTVNFHVKNVMKKLGAANKTQAVAKAAMLGLL

>node166

SAESEEQVFEQIAAVARALGFEYCAYGRVPLPLSKPKVVILSGNYPEAWQQRYMEQGYLE

IDPTVRHGMRSQMPVVWSDELFSSAPETEDRRFWEEARSHGLRHGWAQSSRDANGTSGML

TLCARSGERYPISAPPEIEYQMQWLANVAHAALYSRLMPEGVKLTSREVEVLRWTADGKT

AGEIATILSISERTVNFHVKNAMKKLGAANKTAAVVKAAMLGLL

>node167

SAESEEQVFEQISAVARALGFDYCAYGRVPLPLSKPKVVMLSGNYPEAWQQRYMEQNYLA

IDPTVRHGMRSQMPVVWSDELFSSAPETEDRRFWEEARSHGLRHGWAQSSRDANGTSGML

TLCARSGERYPISEPPEISYQMSWLANVAHAALYSRLMPEGVKLTSREVEVLRWTADGKT

AGEIATILNISERTVNFHIKNAMKKLNAANKTAAAVKAAMLGLL

>node168

SAESEEQVFETISSVARALGFDYCAYGRVPLPLSKPKVVMLSGNYPEAWQQRYMEQNYLA

IDPTVRHGMRSQMPVVWSDELFSSAPETEDRRFWEEARSHGLRHGWAQSSRDANGTSGML

TLCARSGERYPISEPPEISYQMSWLTQVAHAALYSRLMPEGVKLTSREVEVLRWTADGKT

AGEIATILNISERTVNFHIKNAMKKLNAANKTAAAVKAAMLGLL

>node169

SAESEEQLFETISSVAKALGFDYCAYGRVPLPLSEPKVVMLNGNYPEAWQQRYMEQNYLA

IDPTVRHGMRSQMPVVWSDELFSSAPETEDRRFWEEARSHGLRYGWAQSSRDANGTSGML

TLCARSDERYPISEPPEISYQMSWLTQVAHAGLYSRLMPEGVKLTSREIEVLRWTADGKT

AGEIATILNITERTVNFHISNAMKKLNAANKTAAAVKAAMLGLL

>node170

SAESEEQLFETISSVAKALGFDYCAYGRLPLPISEPKVVMLNGNYPEAWQQRYMEQNYLK

IDPTVRHGMRSQMPVVWSDELFSSAKSTEDRRFWEEARSHGLRYGWAQSSRDANGTSGML

TLCARSDERYPISEPPEISYQMSWLTQVAHAGLYSRLMPEGVKLTSREIEVLRWTADGKT

SGEIATILNITERTVNFHISNSMKKLNATNKTAAAVKAAMLGLL

>node171

SAENEEQLFETISSVAKALGFDYCAYGRLPLSISEPKVVMLNGNYPEAWQQRYMEQNYLK

IDPTVRHGMRSLMPVVWSDELFSSAKSTEDKNFWEEARSHGLRYGWAQSSRDAIGTRGML

TLCARSDERYPISEPPEISYQMSWLTQVAHAGLYSRIMPEGVKLTSREKEVLRWTADGKT

SGEIATILNITERTVNFHISNSMQKLNATNKTSAAVRAAMLGLL

>node172

VVKNEYQLFEIVKSTASRLGFDYCAYGQSPLSIAEPKTIMLNGNYPEAWQKRYVERQYVK

IDPTVQHCMVSLQPLVWSDELSQSAKSQAEKDFWEEARSYGLNVGWAQSSRDFIGTRGML

TLCARSNDRYQLSEPPAQYTNMYWLTQTVHSSIYAKIFAKGLYLTNREKEALRWTAEGKT

SAEIAQILGVTERTVNFHLSNSMQKLNVNNKISAAIRAVMLGLL

>node173

SAESEEQFFQTLSSVAKALGFDYCAYGRVPLPLSEPKTVMLNGNYPTAWQQRYQEKNYLA

VDPTVHHGMRSQMPVVWSDELFSSAPETEDRRLWEEARSHGLRYGWAQSSRDANGTAGML

TLCARSDERYPISEPPEISYQMSWLTQVAHAGLYSRLMPEGVKLSSREIEVLRWTADGKT

AGEIATILNITERTVNFHISNAMKKLNAANKTAAAVKAALLGLL

>node174

SAESEEQVFEQIAAAARALGFEYCAYGRVPLPLSKPKVVILSGNYPEAWRQRYMEQGYLE

IDPTVRHGMRSQMPVVWSDELFSSAPETEDRRLWEEARSHGLRHGWAQSSRDANGVSGML

TLCARSGERYPISAPPEIEYQMQWLANTAHAALYSRLMPEGEKLTSREVEVLRWTADGKT

AGEIATILSISERTVNFHVKNAMKKLGAANKTAAVVRAAMLGLL

>node175

SASSEEQVFEQIEAAARALGFEYCAYGRVPLPLSKPKVVILSGNYPEAWRQRYMEAGYLE

IDPTVAHGMRSQMPVVWSDELFSSAPETEDRRLWEEARSHGLRHGWAQSSFDANGVSGML

TLCARSGERYPISAPPENEYRMQWLANTAHAALYSRLMPEGEKLTNREVEVLKWTADGKT

AGEIATILAISENTVNFHVKNAMKKLGAANKTAAVVRAAMLGLL

>node176

SASSEEEVFEQIEAAARALGFEYCAYGRVPWPLSRPKIVILSGNYPEAWKQRYMEAGYLD

IDPTLAHGRRSQMPVVWSDALFSSAPETEDRRLWEEAQSHGLRHGWAQSSFDAYGVSGML

TLCARSRERYPVTAPPENEYRMRWLVNTAHAALYSRLMPDGRGLTDREVEVLKWAADGKT

SGEISTILAISVDTVNFHVKNAIKKLRTANKTAAVVRAAMLGLL

>node177

SASSESQVFEQIEAAARALGFEHCAYGRVPLPLSKPKVVILSGNYPEAWRQRYMEAGYLQ

VDPTVAHGMRSQAPVVWSDELFSDAPETEDRRLWDEARSHGLRVGWAQSSFDANGVSGML

SLCARSGERYPLSAPPENERRMQWLANTAHAALYSRLAGEGEKLTNREVEVLKWTADGKT

AGEIATILAISKNTVDFHVKNAMKKLGAANKTAAVVRAAMLGLL

>node178

RAETPEQLFEQIAAVARALGFEYCAYGRVRLPLSKPKVLIVSGNYPEAWQQRYQEQGYAE

IDPIVRRAKSSQMPVVWSDELFSSQPETEDRAFWEEAASFGLRHGWSQSSRDAAGTFGVL

TLCARSGERYPIDAPPEIEYQLQWLANVAHAALYSRLMGEGVKLTSREREVLRWTADGKT

AGEIAQILSISERTVNFHVKNVMAKLGAANKTQAVAKAAMLGLL

>node179

RAETPEQLFEQIAAVAAALGFEYCAYGRVRLPLSKPKVLIVSGNYPEAWQQRYQELGYAE

IDPIVRRAKSSQRPVVWSDELFSSQPETEDRAFWEEAASFGLRHGWSQSSRDAAGTFGVL

TLCARSGERYPIDAPPRIEYQLQWLANVAHAALYSRLMGEGVKLTSREREVLRWTADGKT

AYEIAQILSISERTVNFHVKNVMAKLGAANKTQAVAKAAMLGLL

>node180

AAESEEQLFEQIAAYARRLGFEYCSYGRVPLPVSKPKVVIFDGTYPDGWMERYQEQGYLE

IDPTVRHGARSQNPIVWSDELFSSAPESEATRLWSDARDFGLKHGVAQSSWAAHGAFGLL

TFCARSGPRYPLTAPPQLRLQLQWLANLAHAAMYSRLAPEGVSLTAREREVLRWTAEGKT

AGEIGQILSISERTVNFHVNNVLEKLGATNKVQAVAKAAALGLI

>node181

AAEDEEQLFEQIAAYARRLGFEYCSYGRVPLPVSKPKVAIFDGTYPDGWMERYQEQGYLE

IDPTVREGARSQNLIVWSDELFISASASEATRLWSDARDFGLKHGVAQSSWAAHGAFGLL

TFCARSGDRYPLTAPPQLRLQLNWLANLAHAAMYSRLAPEGVSLTAREREVLRWTAEGKT

AGEIGQILSISERTVNFHVNNVLEKLGATNKVQAVVKAVALGLI

>node182

AAEDEQQLFQQIAAYARRLGFEYCCYGRVPLPVSKPAVAIFDGTYPDGWMEHYQEQGYLE

IDPTVREGARSSNLIVWPDVDRIEASASEATRLWSDARDFGLNVGVAQSSWAAHGAFGLL

TICARHADRYPLTAPPQLTLQTNWLANLAHALMYSRFAPEGVALTAREREVLCWTGEGKT

ACEIGQILSISERTVNFHVNNVLEKLGATNKVQAVVKAIAMGLI

>node183

AAEDEQQLFQQIAAYSKRLGFEYCCYGRVPLPVSKPAVAIFDGTYPDGWMEHYQEQNYLE

IDPTVREGALSSNMIVWPDVDRIEASASEATRLWSDARDFGLSVGVAQSSWAARGAFGLL

TICARHADRYRLTSPPQLTLQTNWLANLSHSLMYSRFAPEGVALTAREREVLCWTGEGKT

ACEIGQILSISERTVNFHVNNILEKLGATNKVQAVVKAIAMGLI

>node184

AAEDEQQLFQRIAAYSKRLGFEYCCYGRVPLPVSKPAVAIFDGTYPDGWMAHYQAQNYIE

IDSTVRDGALSTNMIVWPDVDRIDPCPSEATRLWQDARDFGLSVGVAQSSWAARGAFGLL

SICARHADRYRLTPPPMLTLQTNWLANLSHSLMYSRFSPAGVTLTAREREVLCWTAEGKT

ACEIGQILSISERTVNFHVNNILEKLGATNKVQAVVKAISAGLI

>node185

AVSSSEQVFALLQKQARRLGFDYFAYGRHPVPFTRPKVFIHSGTYPEAWMERYQEQNYYA

IDPVIQHGLRSQKHVVWSDELFSSARETEDRRLWEEARDFGLRHGVTQSIRAPNGALGIL

SVCARDRPRYSISSPPEIELRLQLLVELLLAALYTRLMSPGVKLSQREREILQWTAEGKT

SGEIAMILSISENTVNFHQKNIQKKFNAPNKTQAAAYAAALGLI

>node186

PIHDSQGVFAVLEKEVRRLGFDYYAYGRHTIPFTRPKTEVHGGTYPKAWLERYQMQNYGA

VDPAILNGLRSSEMVVWSDSLFDQSRMTEDRRLWNEARDWGLCVGATLPIRAPNNLLSVL

SVCARDQQRYNISSPPEIRLRLRCMIELLTQKLYTDLMSPGVCLSHREREILQWTADGKS

SGEIAIILSISESTVNFHHKNIQKKFDAPNKTLAAAYAAALGLI

>node187

AVSSSEQVYALLQQQAQRLGFDYFALCRHPVPFTRPKVFLHSGTYPEAWMEHYQEQNYYA

IDPVLQPGLRSQKHVVWNDELFSSAQETEDRRLWEEARDFGLRHGVTQSIMAPNRALGIL

SVCSRKRPRYSSSSPPEIELRLQLLVELSLAALYTRLMMPGMKFSQRELEILKWTAEGKT

SAEIAMILSISENTVNFHQKNMQKKFNAPNKTQIACYAAAIGLI

>node188

AVSSTEQVTALLQQQLQSLGFDYFALFRHPVPFTRPKVFLHSGTYPEAWMEHYQEQNYYA

IDPVLQLCLRSGKHVVWNDELFSSAQETEDRRLWEEARNFGLLHGLSCSVMASNRAVGIL

SVCSSKRPRYSSSSPPEIELKLQFLVELSLAALYTRLMMPGMDFSQRELEILKWTAEGKT

SAEISLILSISENTVNFHQKNMQKRFNAPNKTQIACYAAAIGLI

>node189

AVSASEQVYALLQQQAQRLEYDYFALCRHPVPFTRPKVSLQTGTYPEAWMSHYQAENYLA

IDPVLKPENFSQGHLPWNDELFSDAQETEDRRLWDAARDHGLRKGVTQCLMLPNRALGFL

SVCSRKRLRYSSSSPPEIELRLQLLVELSLAALYTRLMMPGMKFSKRELEILKWTAEGKT

SAEIAMILSISENTVNFHQKNMQKKFNAPNKTQIACYAAATGLI

>node190

AVSASEQVYALLQQQAQRLEYDYFALCRHPVPFTRPRVSLQTGTYPEAWMSHYQAENYLA

IDPVLKPENFSQGHLPWNDELFSDAQETEDRRLWDAARDHGLRKGVTQCLMLPNHAQGFL

SVCSRKRLRYSSSSPPEIELRLQLLVELSLLALYLRLVMPGMKFSKRELEILKWTAEGKT

SAEIAMILSISENTVNFHQKNMQKKFNAPNKTQIACYAAATGLI

>node191

EMAAAEEVYSELQRQAQRLEYDYYALCRHPVPFTRPKVSFQTGTYPEAWVSHYQAENYLA

IDPVLKPENFSQGHLPWNDDLFSDAQATEDRRLWDAARAHGLRKGVTQCLMLPNRALGFL

SVCSRASLRYRSSPPPEVELRLQLLVRESLAALYTRLMAPGMKFSKREKEILKWTAEGKT

SAEIAMILSISENTVNFHQKNMQKKFNAPNKTQIACYAAATGLI

>node192

RMEAAEEVYHEIELQAQQLEYDYYSLCRHPVPFTRPKVAFYTGNYPEAWVSYYQAKNFLA

IDPVLNPENFSQGHLMWNDDLFSEAQPTEDRRLWEAARAHGLRRGVTQYLMLPNRALGFL

SFCSRCSARYREIPPPELQLKMQLLVRESLMALYMRLMTTGMNFSKREKEILKWTAEGKT

SAEIAMILSISENTVNFHQKNMQKKINAPNKTQVACYAAATGLI

>node193

RMETAEEVYHEIELQAQQLEYDYYSLCRHPVPFTRPKVAFYTGNYPEAWVSYYQAKNFLA

IDPVLNPENFSQGHLMWNDDLFSEAQPTEDRRLWEAARAHGLRRGVTQYLMLPNRALGFL

SFCSRCSARYREIPPPELQLKMQLLVRESLMALYMRLMTTGMNFSKREKEILRWTAEGKT

SAEIAMILSISENTVNFHQKNMQKKINAPNKTQVACYAAATGLI

>node194

EMAAAEDVYSELQRQAQRLEFDYYALCRHPVPFTRPKISFQTGTYPEAWVSHYQAENYLA

IDPVLKPENFSQGHLPWNDDLFNDAQATEDRRLWDAARAHGLRKGVTQCVMLPNRALGFL

SVCSRASLRYRSSPPPEVELRLQLLVRESLAALYTRLMAPGMRFSKREKEILKWTAEGKT

SSEIAMILSISENTVNFHQKNMQKKFNAPNKTQIACYAAATGLI

>node195

EMAAAEDVYTELQYQTQRLEFDYYALCRHPVPFTRPKISLRTGTYPPAWVTHYQSENYFA

IDPVLKPENFRQGHLHWDDVLFHEAQATEDRRMWDAAQRFGLRRGVTQCVMLPNRALGFL

SFCSRASLRYRCSSPPEVELRLQLLARESLSALYTRFMAPGMRFSKREKEILKWTAEGKT

SSEIAIILSISENTVNFHQKNMQKKFNAPNKTQIACYAAATGLI

>node196

QASSEEELFELLAKLARQLGFDHFAYGKVPTSLSKPSVFILSGNYPEEWMERYDEQGYYK

VDPVVQHCMRSTLPLVWNDELFQSKREKQQRRVMEEAKEFGLRNGLTVPIHGPNGRFGML

SFCASDRPEYSAEAPPEAMAELQLLANYLHEAVYQRLQPQGPKLTARERECLQWAAEGKT

SWEIAQILGISERTVNFHLKNAMRKLGATNRQQAVAKAISLGLI

>node197

QASSEEELRELLAKLARQLGFDHFAYGKVPTLLSAPSVFIFSGNFPSEWMERYDEPGYDK

VDPVVQHCMRSTLPLSWNDELFQSKREKQQRRVMELAKEHGLRNGLTVPIHSPNGRFGFL

SFCASDRPEYSAEPPPEAMAEALLLANYLQEAVYERLQPQGPKLSARERECLRWSAEGKT

SWEIALILGISERTVNFHLKNAARKLGVTNRRQAVARAISLGLI

>node198

QASSEEELFELLAKLARQLGFDHFLYGKVPTSLSKPSVFILSGNYPEEWRERYDEQGYYK

VDPVVQHCMRSTLPLVWNDELFQSKREKQQREVMEEAKEFGLRNGLTVPIHGPNGEFGML

SFCASDRPEYSAEAPPEAMPELQLLANYLHEAVYQRLQPQGPKLTARERECLQWAAEGKT

SWEIAQILGISERTVNFHLKNAMRKLGATNRQQAVAKAISLGLI

>node199

QASSEEEWSELLFKLARELGFDQILYGKVPTSHSKSSAFIISGNYPAEWRERYDANGYYK

VDPVVSHCMRSTLPLVWTPEIFQSKREQQQRELYEEASASGLRSGLTMPIHGPRGEFGML

SFCASDRPEYPAEAPPEALPELSLLRDYLLESSYQRLQVQGPKLTARELECLQWVAVGKT

SWEIARILNCSEATVNFHLANVMRKFGATSRQQAVVKAISLGLI

>node200

QASSEEEWSELLFKLARELGFDQILYGKVPTSHSKSSAFIISGNYPAEWRERYDANGYYK

VDPTVSHCMRSTLPLVWTPSIFQSKREQQQRELYEEASASGLRSGLTMPIHGPRGELGML

SFCASDRPEYPAEAPPEALPELSLLRDYALESSYARLQVQGPKLTARELEVLQWVAVGKT

SWEIARILNCSEATVNFHLANVRRKFGVTSRQQAVVKAISLGLI

>node201

RSSGKMEWSAILQKMASDLGFSKILFGLLLPSDSQDNAFIVGGNYPAAWREHYDRAGYAR

VDPTVSHCTQSVLPIFWEPSIYQTRKQHEQHEFFEEASAAGLVYGLTMPLHGARGELGAL

SLSVEAENEYRAEAPPSVLPTLWMLKDYALQSGYAGLPVSGVVLTSREKEVLQWCAIGKT

SWEISVICNCSEANVNFHMGNIRRKFGVTSRRVAAIMAVNLGLI

>node202

NASSEEELFELLAKLARQLGFDHFLYGKFPTSLSKPSVFILSGNYPEEWRERYDEQGYLK

VDPVVQHCMRSTLPLIWNDELFQSKREKQSREVMEEAKEFGLVNGLSFPIHGANGEFGML

SFCATDRPEHSAEAPPEAMPELQLLASYLHEAVYQRLQPQGPELTARERECLQWAAEGKT

SWEIAKILGISERTVTFHLKNATRKLGATNRQQAVAKAISQGLI

>node203

NASSEEELQELLAKLARQMGFDHFLYGKFPTSLSKPSVFILSGNYPEEWREHYDEQGYLK

VDPVVQHCMRSTLPLIWNDELFQSKREKQSREVMEEAKEFGLVNGLSFPIHGANGEFGML

SFCATDRPEHSAEAPPEAMPELQLLASYLHEAVYQRLQPQGPELTARERECLLWAAEGKT

SWEIAKILGISERTVTFHLKNATRKLGATNRQQAVAKAISQGLI

>node204

NADSVEELQELLAKLARAMGFDHFLYGKFPTSLSKPSVFILSGNYPEEWREHYDEQGYLK

VDPVVQHCMRSTLPLIWNDELFQSKREKQSREVMEEAKEFGLVSGLSFSIHGARGEFGML

SFCATDRPEHSREAPPEAMPELQLLASYLHEAVYQRLLPQGPELTARERECLLWAAEGKT

SWEIAKILGISERTVTFHLQNATRKLGATNRQQAVARAISQGLI

>node205

NASSEDELQELLEKFARQMGFDYFLFGIFPTSLSKPDVFILSGNYPEEWREHYDEQGYLK

VDPVVQHCMRQTLPIFWNDELFQSKREKQSREVMEEAKEFGLVNGLSFPIHGANGEFGML

SFCATDRPEHSSEAPPEAMPFLQLLASYLFEAVYQRLQEQGTELTDREKECLFWAAEGKT

SWEIAKILGISERTVTFHLNNATRKLGATNRQQAVAKAISQGLI

>node206

NASDEDELQELLEKFSRQMGFDYFRFAIFPTSMQKPDVVIFNGNCPESWVEAYTEQGYLA

VDPVVQLAMKQTLPIFWNDELWQAKAEKQSREVMELAAEFGLCNGISFPLHGANGEFGIL

SFCTTDRPEHSSELPPEAVPFLSWLASYIFEAVYQRLQEQGTELTDREKECLFWAAEGKT

SWEIAKILGISERTVTFHLNNVTRKLGATNRNQAIAKAISQGII

>node207

NASDEDELQELLERFSRQMGFDYFRFAIFPISMQKPDVVLFNGNCPESWVEAYTEQHYLA

VDPVVQLAMKQTLPIFWNDEPWQAKAEKESREVMELAAEFGLRNGISFPLHGANGEHGIL

SFITTERPEHSSDLPPESVPLLSWLASYIFEAAYLRLQEQGTELTDREKECLFWASEGKT

SWEIATILGISERTVNFHLNQVTRKTGSTNRNQAIAKAISSGII

>node208

TAADERTIVTLLRELSGRMGFDYFRLAIFPSTIQRPDVIIFNGGCPQAWVDTYTSSGFFA

IDPIVKCAMTRSTPILWADEVRNEECDEQGREVMQLASEYGICDGITLPWHGANGHVGLL

SFCITSTPRTSQQWPPSAVPFISWLSMHIFEAVYARVGLSGDALSLRELEVCRWAAEGKQ

VSDIAQILGITPRTVTFHLNNVVSKLGASSKSQAISWALKQGMV

>node209

NASSEDELQELLEKFARQIGFDYFLFGIYPTSLSKPDIFILSGNYPEEWREYYDEQNYLK

VDPVVQYCMSQTLPIHWNDELFQSKREKQSREVMEEAKEFGLVNGLSFPIHSANGGFGML

SFCATDEKEHSSEAPPEAMPFLQLLASYLFEKYYQRLKEQGTELTDREKECLFWACEGKT

SWEISKILGISERTVTFHLNNATRKLGATNRQQAVAKAILQGLI

>node210

ACRSNNDINQCLSDMTKMVHCEYYLLAIYPHSMVKSDISILDGNYPKKWRQYYDDANLIK

YDPIVDYSNSNHSPINWNENFANNKKNKKSPNVIKEAKTSGLITGFSFPIHTANNGFGML

SFCAHSEKEHDNYIPPHACMNIPLIVPSLVDNYYRKINNKGNDLTKREKECLAWACEGKS

SWDISKILGCSERTVTFHLTNAQMKLNTTNRCQSISKAILTGAI

>node211

NVSSQDELQELCEKFARQIGIDYFLFGIYPTSLSKPDIFVLSGNYPEEWREFYHEQNYQR

VDPVVSYCMSQTLPIHWNKELFQSKREKQSRVVMEEAKEFGLVNGLSIPIRSASGGFGML

SLCATDEKEHSSEAPPEAMPFAQLLASHLFEKYYQRLKEQGIELTDREKECLFWACEGKT

SWEISKILGISERTVLFHLNNATRKLGATNRQHAVAKAILQGLI

>node212

NVSSRDELEEVCERFCQLIGIPYYLFGIEQTSLYSPTIHVLSGNYPEEWLEFYFEQNKQR

VDPVVSYIMTQQSPIRWDREEFQSPQQKEGRVVMEKAKEYGLSNGLSIPIRSASGDFALL

SMCAIDEKEDSSEKPPEAMPFAHTLASHLFERYYLRLKEHGIELTQRETECLFWACEGKT

AWEISKIINVSERTVLFHLNNATKKLGATNRQHAVALAIKKGLI

>node213

QASSAEDLKELLAKLANQFGFDHFAYHAVPLSSSAPSPFVLSGNYPDEWVERYLEQGYYK

IDPVVRHCRRSTLPFAWSDEQSQSKLSKEQRRFFEEAREFGIRNGLTIPIHGPNGRFAML

SLCASDRPPYSAEAPPEAMAELQLLAIYFHERVYLKLQSQGVKLTPRERECLQWAAEGKT

SWEIAQILGISERTVKFHLKNARRKLGATTRTQAVAKAISLGLI

>node214

QASDAADLKELLAKLANQFGFDHFAYHAVPLSSSAPSPFVLSGNYPDEWAERYLEQGYYK

IDPVVRRCRSSQLPFAWSDEQSPSKLSKEQRRFFEEAREFGIRNGLTIPIRGPHGRFAML

TLCASDRPPYSAEAPPEAMAALQLLAIYFHARVYLKLQSQGVPLTPRERECLRWAAEGKT

SWEIAQILGISTRTVKFHLENARKKLGASTRTQAVAKAISLGLI

>node215

QASDEADLKEALAKLANQFGFDRFAYLAPPLSSSAPSPKVLSGNYPPEWAERYLEQRYEK

IDPVVRRARSSQRPFAWSDEQTPGKLSKEQRRFFEEAAEFGIRSGLTIPIRGGFGRFAML

TLCASDRPPYSAEAPPEAMAALQLMAIYFHARVYLKLQSQGVPLTPRERECLRWAAEGKT

MWEIAQILGISTRTVKFHLENARKKLGASTLTQAVAKAISRGLI

>node216

QASDEADLKEALANLANQFGFDRFAYLAPPLHVSAPSPKVLSGNYPPEWQERYLEQRYEA

IDPVVTRAKSRKKLFAWSDEQTPGRLSKEQRRFYAEAADFGIRSGITIPIRGGFGRMAML

TLCASDRPPYSIDAPPPAMAALALMQIHFHMSMYLELQSQGVPLTPRELTCLRWSAEGKT

MSEIADILGISARTVKFHLENARKKLGASTLTQAVAIAISRGLI

>node217

QASDEADLKEALANLANQFGFDRFAYLAPPLHVQAPSHKVLSGNYPPEWQERYLEQRYEA

IDPVVTRAKSRKKLFAWSDEQTPGRLSKEQRRFYAEAADFGIRSGITIPIRSGFGRMAML

TLCASDKPPYSIDAPPPAMAAAAVGQIHARMSMYLELQSQGVELTPRELTCLRWSAEGKT

MEEIADIEGISARTVKFHLENARKKLGASTLTQAVAIAISRGLI

>node218

LASDEAALKEALADLANQFGFDGYAYLAPPLHVQAPSHKVVSGNYPPEWQARYLEQKYEA

IDPVVTRAKSRKKVFAWSDEQTPGRLSKEQRRFYAEAADFGIRSGITIPIRTAFGRMSML

TLCASDKPPYSIDLPPPVAAAAAVGQLHARMSMYLELQSQGVELTPKEATCLRWSAEGKT

MEEIADIEGISYRTVKFHLENARKKLDASTLAQAVALAIRRGLI

>node219

LQGDEGILKQALADLADQFGFSGYAYLAPPLHIQRPGHTVVSSNYHPEWRSTYFEKKFEA

VDPVVKRAKSRKQVFTWSGEQERPRLSKEERAFYAQAADFGIRSGITIPIRTANGSMSMF

TLTASDKPPYAIDLPPAVAAAAAVGQLHARMSFYLQLTAQGAWLDPKEATYLRWIAVGKT

MEEIADVEGVKYNSVKVKLEEARKRLDVRTMAHLVALAIRRGLI

>node220

IQGDECILKQGLADLADHFGFTGYAYLAPPLHIQHQHTTAVTSNYHREWRSTYFEKKFEA

VDPVVKRAKSRKHVFTWSGEQERPRLSKEERAFYAQAADFGIRSGITIPIKTANGSMSMF

TLTASDKPPYAIDLPPAVAAAAAVGQLHARISFYLQTTAEGAWLDPKEATYLRWIAVGKT

MEEIADVEGVKYNSVRVKLREAMKRFDVRSKAHLTALAIRRKLI

>node221

LQGDEGILKQALADLADQVGFSGYAYLAPPLHYIRPGHTVASSNYHPEWRSTYFKRKFEA

VDPVVKRAKSLKQVFTWSGEQERPRLSREERAFYAQAADFGIRSGITIPIRAANGSMSMF

TLTASDKPPYAIDLPPAVAAAAAVGQLHTRMSFYLPLTAQGAWLDPKEATYLRWIAVGKT

MEEVADLEGVKYNSVKSKLEETRKRLDVHTMPHLVALAIRAGLI

>node222

VAQDERSIRSALKNFTIASGFDRFAYLAPPLHVSAGDAKTLTGDYPPEWQDIYLEKRYSV

IDPVVTTAKRRKKLFAWSEDQTPGRGSKEQRRFYSEAADFGIRSGITIPIRGGFGRTAML

TLCASDRRPYQIDAPPPAVAALALMHIHFHLSMYLAALSTGVPLTPRELTCLRWSSKGKT

MSEIADLLGISARTVQFHLDNARAKLGASNLTQAVAIAMDRGLI

>node223

VAQDERSIRSALKNFTIASGFDRFAYLAPPLHVSAGDAKTFTGDYPPEWQDIYLEKRYSV

IDPVVTTAKRRKKLFAWSEDQTPGRGSKEQRRFYSEAADFGIRSGITIPIRGSFGRTAML

TLCASDRRPYQIDAPPPAVAALALMYIHFHLSMYLAALSTGVPLSPRELTCLRWSSKGKT

MSEIADLLGISARTVQFYLDNARAKLGASNLTQAVAIAMDRGLI

>node224

VAQDERTIRSALKSFTIACGFDRFAYLAPPLQTSGGEVKTFNGSYPPEWQDIYLANRYSR

IDPVVTTAKRRKKLFVWSADQWPARGSAEQRRFQSEAIDFGIRSGITIPVEGSFGSTLML

TLCASSRRPYQVDAPPPAVAARALMYVHYHLRMYLAALSTGVLLSPRELVCLKWSSKGKT

MSEIADLTGISARTVQYYLDNARAKLEASNLTQAVAIAKDRGLI

>node225

QASDEADLKEALAKLAARFGFDRFAYLAPPLSSSAPSPKVLSGNYPPSWAERYLEQRYEK

IDPVVRRARSSQRPFQWSDEQTPGKLSAAQQRFFEEAAKFGIRCGLTIPIRGGFGRFAAL

TLCASDRPPYSAEAPPEAMAALQLMAIYFHAHVYLKLQSLGVPLTPRERECLRWAAEGKT

MWEIAQILGISTRTVAFHLENARKKLGASTLTQAVAKAASRGLI

>node226

QSVDEADLREALAKAAARFDFPRFAYLLPSLSSSAPKPKVLSGNYPPSWAERYLEQRYEK

LDPVILRARNGGRPFQWGSDLTRGKLSAAQQRLFEEAARFGIRCGLTIPIRDHRGRFAAL

TLCASDQPPYRPELPPRYEQALQLMAICFHRHVYLKLRTVGVLLTPREYECLRWAAEGKS

AWEIAQILGISTRTVAFHLENAKKKLGVRTLNQAVARLASRGLI

>node227

QASSAEDLKELLEKLANQFGIDHFAYHAVPLSSSAASPFVLSGNYPDEWVERYLEQNYYK

IDPVVRHARRSILPFAWSDEQSQSKLSKEQREFFKEAREFGINNGLTIPIHGPNGRFAML

SLCNSDRPPYSAEFPPEAMGDLQLLAIYFHERVYLKLQSQGAKLTPRETECLQWAAMGKT

SKEIAEILGISERTVKFHLKNARRKLGATTTTQAVAKAISLGLI

>node228

QASSAEDLKELLEKLRDQFGIDHVAYHAVPLSSSAASPFVCTGTYPDEWVERYLEQNYYK

IDPVVRQARRSILPVDWSDSQWQSKRSKEAREFFKEAREFGINQGLTIPIRGPNGRFALL

SVCNSDCPPYDAEWPPEAMGDLQLLAIYFHERVYLKLQSAGPKLSPREIECLQWTAMGKS

SKEIADILGISERTVKFHLKSARRKLGAATTTQAVARAISLGLI

>node229

QASSAEDLEALLEKLRDQYGIDHVVYHAVPLSSSAASPFVCTGTYPDEWVERYLERNYYK

IDPVVRQARRSFLPVDWSDSQWQSKRSKEAREFFKEAREFGINQGLTIPIRGPNGRFALL

SVCNSDCPPYDAEWPPESMRDLQLLAHYFHERAYLELQSAGPKLSPREIECLQWTAMGKS

SKEIADILGISERTVRFYLKSARRKLGAATTTQAVARAISLGLI

>node230

QASSAEDLEALLEKLRDEYGIDHVVYHAVPLSSSAASPFVCTGTYPDAWVERYLERNYYK

IDPVVRQAFQRFLPVDWSDSQWQSKVSKEAREFLKDAREHGINQGYSIPIRGPNGRFALL

SVCNSDCPPYDDEWPPEYRRDLILLAHYFHRRAYLELQSAGPKLSPREIECLQWTAMGKS

SKEIADILGISEHTVRFYLKSARHKLGAATTTQAVARAISLGLI

>node231

AASSLEDLEALTEKLRDEYGIDHVVYHAVPWWVSAAGQYYCAGTYPKAWVERYLERNYHR

IDPVILGCFQRFHPVDWKRLDWSGKVAKAAREFLRDARAHGLNQGYSIPIRGPNGQFALF

TVCNHDCDPYDDAWPPEYRRDLILLAHYFNRKAYLEFQPAGPNLSPREIDAMTLLAMGYS

RAQVADTLSISEHTLRVYIESARHKLGALNTTHAVARALSRGLI

>node232

AASSLEDLQALTERLRDEYGVGHVVYHAVPWWVNAAGQQYGAGTYPKAWVERYLERNYLR

IDPVILGCFQRFHPVDWKRLDWSGKAARAAREFLRDARAHGLNQGYSIPIRGPNGQFALF

TVCNHDCDPYDDAWPPENRRDLILLAHYFNRKAYLEFQPAGPSLSPREIDAMTLLAMGYS

RAQVADTLSISEHTLRVYIESARHKLGALNTTHAVARALSRGVI

>node233

QIKAAANVDAALRILQAEYGLDFVTYHALTIASKVDSPFVRTGTYPDAWVSRYLLRSYVK

IDPIVRQGFERYLPFDWSDSQWEVEVSPEAHEFLVDAREHGIGNGYSIPIADKAQRRALL

SVCNSRVPPYPDEWPPQYRNEWIELAHLIHRRAVVELENDGPQLSPREIECLHWTALGKD

SKDIATILGISEHTTRSYLKSARHKLGAATITAAASRAIQLRLI

>node234

QASSAEDLEALLEKLRDQYRIDHLVYHAVPLSSSAASPFVCTGTYPDEWVERYLERNYYK

IDPVVRLARRGFLPVDWSDSQWQSKRSCEAREFFKEAREFGINQGVTIPIRGPNGRFALL

SVCNSNLPPYDAEWPPESMRDLQLLAHYFHERAYLELQSAGPKLSPREIECLQWTAMGKS

SKEIADILGISERTVRFYLKSARRKLNAATTTQAVARAISLGLI

>node235

QASSAEDLEATLEKLRDQYRISHLVFHAVPLSSSAASPFVCTGTYPDEWVERYLERNYFK

IDPVVRLARRGFLPVDWSDSQWQSKRSCEAREFFKEARAFGIRQGVTIPIRGPNGERSLF

SVCTSNLPPYDAEWPPESMRDLQLLAHYLHERAYLELETAGPKLSRRELECLQLTARGLL

SKQIAARLGISESAVRLYLKSARRKLNAATTSQAVARATSLELI

>node236

LPASTDPFREFLDELRDQFELDHVAYAANPISSAAQGHMGYVGTYPDEWTAHYLEQGYYE

IDPTLHKARRSIAPVDWSDSQWRLERSNEFRTIFRDARDFGIDRGLTIPIRGPYGDVGLL

SVCNRDCSPYDREWPPEVIGDLQSAAVHMHDTVYLRIRMLGPSLSTREIEILQWTAAGKS

QQDIGDILSISHRTVEVHLRSARQKLSALTTPQAVGRAISLGLI

>node237

LPASTDPFREFLLELRDQLELDHAAYAANPISSAAQGHMGYVGTYPDEWKAHYLEQGFHE

IDPTLHKARRSIAPVDWSDSQWRLERSNDFRTIFRDARDFGIDRGLTIPIRGPYGDVGLL

SVCTRDCSPYDREWPPEVIGDLQSAAVHMHDTVYLRIRMLGPSLSTREIEILQWTAAGKS

QQDIGDILSISHRTVEVHLRSARQKLSALTTPQAVGRAISLGLI

>node238

NESAPEDLKQLLEKLANEFGIGHFAYAAMMDKSSLAQPFILSGNYPDEWVETYIENNYHL

IDPVIRHALHSITPFSWSDEQNQAQLGEEQSEFFKQAREFGINNGYTFTVHGPNGYFAML

SLCNSDRQPESNDFPPEKKGDLQMLLISFHERVYLKLQPNGAKLTPRETEVLKWAAMGKT

YKEIAEILGISERTVKFHMSNVVRKLEVTTAKQAVSKAIKLGLI

>node239

NEGAPEDLKQLIEKLANEFGIGDFAYAAMMDKSSLAQPFILSGNYPDEWVETYIENNYHL

IDPVIRHALHSITPFSWSDEQNQAQLVEEQSEFFKQAREFGLNNGYTFTVHDPNGYFAML

SLCNSDRQPESNDFPPEKKGDLQMLLISFHERVYLKLPPNGAKLTPRETEVLKWAAMGKT

YSEIAEILGISERTVKFHMSNVVRKLEVTTAKQAVSKAIKLGLI

>node240

NESMPEDIKQLLEKKLNEYGIGHFAYAAMMDKSSLSQPFIISGNYPQEWVETYIENNYHL

IDPVIRHALNRITPFSWSDENNLANLGEEQSEVFKQAREFNINNGYTFVVHGHNGYFAVL

SLCSSDRQPESNDFPPEKKGDLQMLLISVHEKAYLKLQPNGAKLTPRETEVLYWASMGKT

YKEIALILGISERTVKFHMSNVVRKLEVTNAKHAISKAIELGLI

>node241

NESMPEDIKQYLEKKLNEYGIGKFAYAAMMDKSSLSQPFIISGNYPQEWVETYIENNYHL

IDPVIRHALNRVTPFSWSDENNLANLGEEQSEVFKQAREFNINNGYTFVVHGHNGNLAVL

SICSSDRQPESNDFPPEKKGDLQMLLISVHEKAYLKLQPNGAKLTPRETEVLYWASMGKT

YKEIALILGISERTVKFHMSNVVRKLEVTNAKHAISKAIELQLI

>node242

NESMNEDIKSYLERKLKEYGNVKFAYAAMMDKSSLSQPFIISGNYPQEWVETYIENNYHL

IDPVIRHALNRVTPFSWSDDNNLANLGSEQSEVFKQAREYNINNGYTFVLHDHNNNLAVL

SICSNDDQPESNDFPPEKKGDLQMLLISVHEKAYLKLQPNGALLTPRETEVLYWASMGKT

YKEIALILGISERTVKFHMSNVVRKLEVTNAKHAISKAIELQLI

>node243

NESINETIKSYLERKLKEYGNIKYAYAAIMNKKNPSQVFIISGNYPQEWVETYKENNYQF

IDPVIITALNRVTPFSWDEDNILINSGSKLSKIFNQAREYNINNGYTFVLHDHNNNLVVL

SIISDDDAPESTDMPPENKGDLQMLLISVHEKMYLTLQQNGALFSPRENEILYWASMGKT

YQEIALILGITTRTVKFHMSNVVKKLGVTNAKHAIRLGVELQLI

>node244

NESINETIKSYLERKLKEYGNIKYAYAAIMNKKNPSQVFIISGNYPQEWVETYKENNYQF

IDPVIITALNRVTPFSWDEDNILINGGSKLSKIFNQAREYNINNGYTFVLHDYNNNLVVL

SIISDDDAPESTDMPPENKGDLQMLLISVHEKMYLTLQQNGALFSPRENEILYWASMGKT

YQEIALILGITTRTVKFHMSNVVKKLGVTNAKHAIRLGVELQLI

>node245

NESINETIKSYLERKLKEYGNIKYAYAAIMNKKNPSQVFIISGNYPQEWVETYKENNYQF

IDPVIITALNRVTPFSWDEDNIVINSGSKLSKIFNLAKEYNIVNGYTFVLHDHNNNLVVL

SIISDDDAPESTDMPPENKDKLQMLLISVHEKMYLTLQQNGELFSPRENEILYWASMGKT

YQEIALILGIKTSTVKFHIGNVVKKLGVTNAKHAIRLGVELQLI

>node246

NESINETIKSYLERKLKEYGNIKYAYMAIMNKKNPSQVFIISGNYPQEWVETYKENNYQF

IDPVILTALNKVSPFSWDEDNIVINSKSKLSKIFNLAKEYNIVNGYTFVLHDHNNNLAVL

SIISDDDAPESTDMPPENKDKLQMLLISVHEKMYLTLQQNGEIFSPRENEILYWASMGKT

YQEIALILGIKTSTVKFHIGNVVKKLGVLNAKHAIRLGVELQLI

>node247

NEIINETIKSYLNRKLKQYGNIKYAYMAIMNKKNPSQVFIISGNYPQEWVETYKENNYQH

IDPVILTALNKVSPFSWDEDNIVINSKLKLSKIFNLSKEYNIVNGYTFVLHDHNNNLAVL

SIIFDDNAPEPTDMPPENKDKLQMLLISVHEKMYLTLQQNGEIFSPRENEILYWASMGKT

YQEIALILGIKTSTVKFHIGNVVKKLGVLNAKHAIRLGVELQLI

>node248

NEIINETIKSYLNRKLNQYGNIKYAYMAVMNKKNPSEVFIISGSYPDEWVELYKENNYQH

IDPVVLTAFNKVSPFSWDEENIVINSKLKLSKIFNLSKKYNIVNGYTFVLHDHNNNLAML

SIIIDDNAPEPTDVPPENKDKLQMLLIDVHEKMYLTLQQNGEIFSPRENEILYWASMGKT

YQEIALILGIKTSTVKFHIGNVVKKLGVLNAKHAIRLGVELQLI

>node249

SEIISRVIKGHFNENLDHYDGIKFSFMAVLNKKNPSEMLIISGSYPDEWVNLYKENKYQH

IDPVVLASFNKISPFSWEEKSLVINTRLQLAKIFDLSKKYNIINGYTFVLHDHGDNLAML

SIIIDDSSPEPDDVPPEKKDTFQMLLIDAYEKIYISLQKNKEIFSQRENEILYWASMGKT

YLEVAIILGIKTSTVKFHIGNVVKKLGVLNAKHAIRLGVELQLI

>node250

NETITETLKSYINRKLNLYGSPKYAYMAVINKKNPSDVFIISGSYPDEWVELYKENNYQH

IDPVVLTAFRRVSPFSWDEENITIMSELKLSKIFTLSKKYNIVNGFTFVLHDHMNNLAML

SIIIDDNAPEQTDVPPNDKDRLQMLLIDVHEKMYLTLQQSGAIFSPRENEVLYWASMGKT

YQEIALITGISTSTVKFHIGNVVKKLGVLNAKQAIRLGVELELI

>node251

NETITETLKSYINRKLNLYGSPEYAYTAVVSKKNPSDVLIISGSYPDEWVELYRENNYQL

TDPVVLTAFRRTSPFSWDEENITLMSELKLTKIFTLSKKYNIVNGFTFVLHDHMNNLALL

SVIIKDNDPEQTDLPPNEKDRMQMLLIDVNEQMYYRLQQSGAIFSSRENEVLYWASMGKT

YSEIALITGISVSTVKFHIGNVVKKLGVSNARQAIRLGVELDLI

>node252

NQTITDTLQTYIQRKLSSFGSPEYAYTAVVSKKNPSNVLIISGSYPDEWVRLYRANNFQL

TDPVILTAFKRTSPFAWDEENITLMSDLRFTKIFSLSKQYNIVNGFTYVLHDHMNNLALL

SVIIKGNDPEQTALPPSEQGTMQMLLIDFNEQMYYRLQNAGTIFSSRENEVLYWASMGKT

YSEIAAITGISVSTVKFHIKNVVVKLGVSNARQAIRLGVELDLI

>node253

QASSAEELFELLAKLARQFGFDHFAYGAVPLPSSKPSSLVLSGNYPDEWVERYLEQGYYK

IDPVVRHCRRSTSPFVWSDEQSQQKLEKQQRRVMEEAREFGLRNGLTVPIHGPNGRFAAL

SFCASDHPPYSAEAPPEAMAELQLLAIYFHERVYRRLQSQGVKLTPRERECLQWAAEGKT

SWEIAQILGISERTVNFHLKNAMRKLGATNRTQAVAKAIRLGLI

>node254

RASSADQLFEQALQLASELGFDALVYDYSPVPFSALSSLLIPRNTPEDWLELWCEQGYYQ

IDPVQQLALRSSSPFVWSAETALQKFTDQHKPVVRYLHDHGMTSGLTVPIHLPKGGFATL

TGCASDGSDVAAEAPPQALAEFSLLAHAFQERAYYPLRSCGVKLTRRERECLQWAAEGLT

SREIAEKLNRSEATVNLHLNSAMRKLGARNRVQAVVRAVHYRLL

>node255

RASTADQLFDQALQLASELGFDALIYDYSPVPFSALISLLKLRNTPEDWHELWCEQGYYQ

IDPVQQLALRSSSPFVWSAETALQKFTDQHKPVVRYLHDHGMTSGLTVPIHLPKGGFATL

TGCASDGSDVAAEAPPQALAEFSLLAHAFQERAYYPLRSCGVKLTRRERECLQWSAEGLT

AREIAEKLNRSVATVTLHLNSAMRKLGARNRVQAVVRAVHYRLL

>node256

RATTADGLFDQAFQLASELGFDALIYDYTPVPFSALISLLKLRNVPEDMHELWCERGYYQ

IDPVQQLALRSSTPFVWSANTALQKFSDQHKPVTRYLCDHGMTSGVTVPIHLPKGGFATL

TGCASDGSDVAAEAPPAALAEFSLLAHAFQERAYYPLRSCGVKLTRRERECLQWSAEGLT

AKEIARKLNRSVATVTLHLNSAARKLGARNRVQAVVRAMHYRLL

>node257

RATTVDGAFDQLFQQTSRLGFDALIYDYTPVPRSELISLLKMCNVPEDMQQLWCERGYYQ

VDPVQHYALESCAPFVWSDNTSLQRLSDNPKPVTHYMCDHNMPSGATVPLHLPNGGFVTL

TGCIHTGQDVGAEHPPAVLAELSLLALTFQESAYFPLLTCGVKLSKRERECLTWSAEGLT

AKEIARKLNRSVATVTLHLNTAARKLGASNRVQAVVRAMHYRLL

>node258

RLTTADGLNDQAFQLVSELGFDALIYDYTPVPFSALISVLKLRNVPEDMHELWCERGYYQ

IDPVQQLALRSSTPFVWSANTALQKYSDQHRPVTRYLCDHGMTSGVTVPIHLPKGGFATL

TGCASDGSDVAAEAPPAALAEFSLLAHAFQERAYYPLRSCGVKLTRRERECLQYSAEGLT

AKEIARKLNRSVATVTLHLNSAARKLGARNRVQAVVRAMHYRLL

>node259

QASTAEELFELLAKVARQFGFDHFAVGAVPLPSSKQSSLVLSGNWPDEWLERYLEQGYVK

NDPVVRHCRQSTSPFVWSDEQSQQKLEKQQRRVMEEAREFGLRNGLVVPIHGPNGLLGAL

SFCASDDPPYGAEAPPSAMAELHLLAIYFFERLYRRLESQGVKLTPRERECLQWAAEGKT

SWEIAQILGISERTVNFHLKNAMRKLGATNRTQAVAKAIRLGLI

>node260

QASTAEELFELLAKVARQFGFDHFAVGAVPLPSSKQSSLVLSGNWPDEWLERYLEQGYVK

NDPVVRHCRQSTSPFVWSDEQSQQKLEKQQRRVMEEAREFGLRNGLVVPIHGPGGLLGAL

SFCASDDPPYGAEAPPSAMAELHLLAIYFFERLYRRLESQGVKLTPRERECLQWAAEGKT

SWEIAQILGISERTVNFHLKNAMRKLGATNRTQAVAKAIRLGLI

>node261

RLSEVTDLAERIAAVGREMGLPYVAVSDSPSPMIGRPLAETTGRWVDPGLAYWRDRAFAL

RAPIIRAVRVTAEPFCYHGKMASWRQARNRIEVAEAAGSFGVRSAIVAPIHSPGGVIGAV

VWCATDDSPYAVDVPPARAAELHMLALRFLSAYYRDAADPGVKLTRREIQCLKWAAAGKT

DSEIAQIMGISAPTVRFHMQNAARKLGVSGRSQAIRRATTLGYI

>node262

QARTAEEVFELLAKVARQFGFDHFAVGAVPLPSSKQSSLVLSGNWPAEWLERYLEQGYVK

NDPVVRHCRQSTSPFVWSDAQSQQGLEKQQRRVMEEAREFGLRDGLVVPIHGANGLLGAL

SFCGGDDPPYGGERPPSAMAELHLLAIYAFDRLYRRLESQGVKLTPRERECLQWAAEGKT

SWEIAQILGISERTVNHHLKNAMRKLGAVNRTQAVAKAIRLGLI

>node263

QARTAEEVFELLAKVARQFGFDHFIVGAVPLPGSKQRSLVLLGNWPAEWLERYLEQGYVH

HDPVVRHCRQSTSPFVWSDAPSQQGLEKQQRRVMDEAREFGLRDGLVVPIHGANGLLGAV

SFCGGDDPPYGGERPPSAMAELHLLAIYAFDRLYRRLESQGSKLTPRERECLQWAAEGKT

SWEIAQILGISERTVEHHLKNARRKLGAVNRTQAVAKAIRRGII

>node264

QARTAEEVMELLAKVARRFGFDHFIVSGIPLPGEKQRSLVLLGNWPAEWFERYLENGYVH

HDPVVRHCRQSTSPFVWSDAPSNQGLEKQSRRVMDEAREFGLRDGLVVPIHGANGLQGAV

SFCGGDDPPYGGERPPSAMAALHLLAIYAFDALRRRLESQGSKLTPREREVLQWAAEGKT

SWEIAQILGISERTVENHLKNARRKLGAVNRTQAVAKAIRRGII

>node265

QARTAEEVMNLLAKVARRFGFDHFIVSGIPLPGEKQRSMVLLNNWPAGWFERYLENNYVH

HDPVVRHCRQSTSPFVWSDAPSNQELEKQSRRVMDEAREFGLSDGFVVPIHGANGFQAAV

SFCGGDDPPYGGERPPRARAALHLMAIYAFDALRRRLESQGSRLTPREREVLQWAAEGKT

SWEIAQILGISERTVENHLKNARRKLGAVNRTQAVAKAIRRGII

>node266

QARTAEEVMNLLAKVARRFGFDHFIVSGIPLPGEKQRSMVLLNNWPAGWFERYLENNYVH

HDPVVRHCRQSTSPFVWSDAPSNQELEKQSRRVMDEAREFGLSDGFVVPIHTANGFQAVV

SFCGGDDPPYGGERPPRARAALHLMAIYAHSALRRRLESQGSRLTPREREVLQWAAAGKT

SWEIAQILGISERTVENHLKNARRKLGAVNRTQAVAEAIRRGII

>node267

SAKSREDVMNLLAKVAHHFGFNYFAISGIPIPSERERSYFMLNNWPAGWFERYLENNYVH

ADPVVHLCRMSDSAFVWSEALRNQELDRQSRRVMDEAREFTLNDGFSVPLHTAGGFQAIV

SFCGGDDPPYGAEKPPRARAALHLMAIYAHSALRRQLKNQGPRITAREREIIQWCAAGKT

AMEIATILGRSHRTIQNEISNVQRKLNVVNAAQMIAESFRAGII

>node268

QARTAEEVMNLLAKVARRFGFDHFIVSGIPLPGEKQRSMVLLNNWPAGWFERYLENNYVH

HDPVVRHCRQSLSPFVWSDAPSNQELEKQSRRVMDEAREFGLSDGFVVPIHTANGFQAVV

SFCGGDDPPYGGERPPRARAALHLMAIYAHSALRRRLESQGSRLTPREREVLQWAAAGKT

SWEIAQILGISERTVENHLKNARRKLGAVNRTQAVAEAIRRGII

>node269

GLRTVPDVMNALEAAFGRFGFETIIVTGLPLPNQRQRQMVLAKRWPAGWFNLYTQNNYDR

FDPVVRLCRQSVNPFEWSEAPYDAELEPSAAEVMNRAGDFRMSRGFIVPIHGLTGYEAAV

SLCGGDDPPYGGVHPPRSKPALHLMAMYGFDHIRRRLAPHGTRLTPREREVISWASQGKS

AWEIGEILHITQRTAEEHLATAARKLGAVNRTHAVALAIRHKII

>node270

QARTAEEVMELLAGVARRFGFDHFIVSGIPLPGEKQRALVLLGNWPAEWFERYEENGYVH

HDPVVRHCRQSTSPFVWSDAPPNQGLEKQSRRVMDEAREFGLRDGLVVPIHGANGLQGAV

SFCGGDDPPYGGERPPSAMAALQALCIYAFRALRRRLESQGSKLTPREREVLQWAAEGKT

SWEIAQILGISERTVENHLRNARRKLGAVNRAQAVAKAIRRGEI

>node271

KARTAEAVCEVLAGIAARLGFDRVIVCGIPLPGEKQRALFLVGNWPGDWFEGRERDGYLH

HCPVTRHVLESDAPFFWSDAPPNQGLERQSYRIVKEARDLGLRDGLQVPVFGANGLEGAV

SFCGGDDPPYAGERPPSAKLALQALCTIAFRALRRRLAAQGSALSPREREVLQWVAAGKQ

QAEIAAILGISERTVENHLRNARRRLGAASTAQAVAKAIRRGDI

>node272

QARTAEEVFEQLLKVASEFGFTHLIAGAVPQPGSTRRSLVLLGNWPAEWLERYLEQGYVY

HDPVVRHLRQETSPFVWRDAPSQIGVEKQQRQVMGEAREFGLRDGLVVPIITADGELGAV

SFCGGDDPPYGGERPPSALGELNLVAIYALGRLYRRLESQGSKLTPRERECLQWAAEGKT

SWEISQILGISERTVEHHLKNARAKLGAVNRAQAVAEAIRRGII

>node273

QARTAAEVCEQLLGVASEFGFTALIAGAVPQPGSTRRSHVLLGDWPAEWLERYLERGYVY

HDPVVRHLRQETSPFVWRDAASQIGVEKSQRQVMGEAREFGLRDGLVVPIITLDGEIVAV

SFCGGDDPPYGGERPPAELGLLNLVATYALGRLYRRLESQGSKLTPRERECLQWAAEGKT

SWEISQILGISERTVEKHLLNARAKLGAVNRAQAVAEAIRRGII

>node274

QARTAAEVADALLGVAAEFGFTALFGGVRPQPLAERRSRILLQEFPAEWLDRYSRRGYVY

RDPVVQHLRHERSPFTWRDEAYESAAQRSVRLIQGEAAEFGLRDGYVVPISTLDGEIVAI

SFCGGDDPPYGGPRPPADLSLLNFAASYALGRLLHRLASRGSKLTAREWDCLLWAAEGKT

NWEISAILGISKSTVTKHILSARAKLGAVSKAHAIAMAIRTKIL

>node275

QTRTAAEIEDCLLGLAAEFGFTGLFGGVRPAPLAELRSRILFQRFPAQWLDRYSRKGYVF

RDPIIQHLFHERTPFTWHDEGYESCPQRSVRLIQGEAAEFGLRDGYVVPISTIDGEIVSL

SFCGGDDPPYGGPGPPADLSVLNFAASYALGRHLHRIWRRGSNLTAREWDCLLWAGEGKT

NWEISAILGISKSTVTKHIMAARAKLGAVSKAHAIAMAIRTKLL

>node276

EARTREEVFELLERVAEQYGFDHFALLAVPSPSSEQASLVLSGNWPAEWLEAYLRQGYVK

NDPVVRHLRQSTSPFVWSDAQSAYGKEKKMARVMEEAREHGLEDGLVFPVHGANGLLGAL

SFGGGDDPPYGGERPPSAMAELHLIAIHAFDRLYAELESRGVKLTPRERECLKWAAEGKT

SSEIAQILGISEHTVNHYLKNAMRKLGAVNRTQAVAKAIRLGLI

>node277

EMRTREEVVAEFERLIEQYGFDYYGLLRAPKPREDPASLLLAGHWPEGWPETYMRKKYVL

IDPTVRYLGHAQRPFRWRDTLSAFRDHKKMERMMVDARQHGLEDGYVFPVHGRRGLLGSL

SLGGGKDPSVGGEEPPSAISRLYWIAIHAFDRLYAELASRGVQLTRREMEALNYLAEGMT

SNDISKVLGISSHTVDWYMNGIQEKLGAKNRHHAVAIAFRLGLI

>node278

EMRTREEVTAEFERLIKQYGFDYYGLLRAPKPVEDPASLLLAGHWPEGWPETYMRKKYML

VDPTVRYLGHAQRPFRWRDTLGAFRDHKKMERMMVDARQHGLEDGYVFPVHGRRGLLGSL

SLGGGKDPSVEVELPPSAARRLYWKLIHAPDRAYAELSSVGVELTRREMEALSLLAEGMT

SNDIGRVLGISSHTVDWYMNGIQEKLGAKNRHHAVAIAFRLGLI

>node279

EMRTREEVVAEFERLIEQCGFDYYGLLRQPKPREDPASLLLAGRWPEGWPQIYMRKKYVL

IDPTVRYLGHAQRPFRWRDTLSAFRDHKKMERMMVDARNHGLEDGYIFPVHGRRGLLGSL

SLGGGKDPSVGGKEPPIEISLFDQIAKRAFWRLYLELASRGVQLTRREMEALNYLAEGMT

SNDISKVLDISSHTVDWYMNGIQEKLKAKNRHHAVAIAFRLGLI

>node280

EARTREDVFELLERVAEQYGFDHFALLAVPSPSSEEASLVITGNWPAELLEAYDRQGLLK

NSPVVRRLRQSTTPFVWDDAQSAYGKEDKMAEVMELLREHGLESGLYFPVHGAAGLRGAL

SFGGGDDPPYGGERPPSAMAELHLIAIHAFDRLYAEIENRGVALTPRERECLKWAAEGKT

SSEIAQILGLSEHTVNHYLTNATRKLDAVNRTQAVAKAIRLGLI

>node281

EAWTREDVFELLERVAEQYGFDHFALLAVPSPSSEEASLVITGNWPAELLEGYDRQGLLK

NSPVVRRLRQSVTPFVWDDAQSAYGKEDKMAEVMELLREHGLLSGLYFPVHGAAGLRGAL

SFGGGDDPPYGGERPPSAMAELHLIAFHAFDRLYAEIENRGVALTPRERECLKWAAEGKT

SSEIAQILGLSEHTVNHYLTNATRKLDAVNRTQAVAKAIRLGLI

>node282

EARTREDVFELLKRVAEQYGFDHFALLAVPSPSSEEASLVITGNWPAELLEAYDRQGLLK

NSPVVRRLRQSTTPFVWDDAQSADGKEDKAAEVMELLRRHGLESGLYFPVHGAAGLRGAL

SFGGGDDPPYGGERPPSEMAELHLIAIHAFDRLYAEIENRGVALTPRERECLKWTAEGKT

SSEIAQILGLSEHTVNHYLTNATRKLDAVNRTQAVAKAIRLGLI

>node283

EARTREDFFELLKRVAEQYGFDHFALLAVHSPSSEEASLVITGNWPAELLEAYDRQHLLK

NSPVVRRLRQSTTPSVWHDADSADGKEAKAAELMELLVRHGLESGLYFPVHAAAGRRAVL

FFGGGDDPPYGGERPPSEMAELHLIAIHAFDRLYAEIENRGVALTPRERECLKWTAEGKT

SSEIAQILGLSEHTVNSYLTNAIRKLDAVNRTQAVAKAIRLGLI

>node284

EARTREDFFELLKRVAEQYGFDHFALLAVHSPSSEEASLVITGNLPAELLEAYDRQHLLK

NSPVVRRLRQSTTPSVWHDADSADGKAAKAAELRELLVRHGLESGLYFPVHAAAGRRAVL

FFGGGDDPPYGGERPPSEMAELHLIAIHAFDRLYAEIENRGVALTPREREVLKWTAEGKT

SSEIAQILGLSEHTVNSYLTNAIRKLDAVNRTQMVAKAIRLGLI

>node285

KALNREDFFRLLKRVAEQYGFDHFALLAVHSESSAEASLVITGNLPAELLEAYDRQHRLK

NSPVVRRLRQSTTPSVWRDADSADGKAAKAADFRELLVRHGFESGLYIPVHAAAGRYAVL

FFGGGDDPPYGGERPPSEMAELHLDAIHAFDRLYAEIENRGVALTPREREILKWTSEGKT

SSEIALILGLSEHTVNSHLTNAIRKLDAVNRTQMVAKAIREGLI

>node286

KALNRVDFFRIFHTLALRYGFDHFGILQVNNENTAHATLVVHHDLPAGLAEAYDKRHRLN

DSAIFKSLHKSTIPSVWRSDDPVESGAAGSADFQELLVQLGFEMALSIPVHAAAGRYAVL

FLGGGDGDPYGNDIPPSEHYELTYDAVCAFDYFYRKIANKGMGLTPRETEILKWISHGKT

ASEIALIVSVSEHTVNSHTATILKKLDVVNRTQMVAKAIREQII

>node287

ELRTQEDVFELLKRVAEQYGFSHFLLLSVPSTSSEEASTVITGNWPAELLNAYDSQGLLK

NSPVIRRLRQSTTPFVWDDAQSADGKEDKAAEVIELLRRHGMERGAYFPVHDAAGLRGAI

SFGGGDDPPYGGERPPSEMAELHLIAIHVFDRLYAEIEKRGVALTPREIECLSWTAAGKT

SAEIAEILGLSEHTVNHYLNRATRKLDAVNRTQAVAKAIRLGLI

>node288

GLRTQYDVFRLLKRVAEQYGFSHFLLLSVPTTSSEEASTVITGNWPAELLNAYDSQGLLK

NSPVIRRLRQSTTPFVWDDAQNADREEDKAAEVIELFRRFGMERGAYFPVHDAAGLRGAI

SFGGGDDPPYSGERPPSEMAELHYIAIHVFDRLYAEIEKRGVALTPREIECLSWTAAGKT

SAEIAEILDLSEHTVNHYLNRATKKLDAVNRTQAVAKALRLGLI

>node289

GLRTQYDVFRFLKRVTEQYGFSHFLLLSVPTTSSVEASTVITGNWPAELLNAYDSQGLLV

NSPVIRRLRQSTTPFVYDDAQNADREEGKAAEVIELFRRFGMERGAYFPVHDAAGLRGAI

SFGGGDDPPYSGERPPSEMAELHYIAIHVYDRLYAEIEKRGVALTPREIDCLSWTAAGKT

SAEIAEILDLSEHTVNHYLNRATKKLDAVNRTQAVAKALRLGLI

>node290

ELRTQEDVFELLKRVAEQYGFRHFLLLSVPSTSSEEASTVITGNWPAELLNAYDSQGLLK

NSPVIRRLRQSTTPFVWDDRQSADGKEDKAAEVIELLRRHGMERGAYFPVHDAAGLRGAI

AFGGGDDPPYGGERPPSEMAELHLIAIHVFDRLYAEIEKRGVALTPREIECLSWTAAGKT

SAEIAEILGLSEHTVNHYLNRATRKLDAVNRTQAVAKAIRLGLI

>node291

ELRTQEDVFEMLDRVAEQYGFRGFLLLSVPSTSSNDSNAILTGSWPAELLKAYDSAGLIN

GSPVIQRLRQSTTPFTYDDRRRADGKADKAAGVFELLRRHGMPRGAYFPVHDAAGLRGAI

AFGGGDDPPYGGERPPSEMAELNLVANLIFSKLYAEIGRRGVSLSRREIECLSWAAAGKT

SAEMAEILGLSEYTVNHYLNRATRKLDAVNRVQAVAKAIRAGLI

>node292

EIATLETQFDYMRRVAEQYGFKHFLILSIPSIDAEKASTVISGNWPAELLNKYDSLSMLR

HSAGIRRLRQTTTPFAWDDRQSAGGKEDSAAELIELLRGHGMLRGSYFPVHDAAGNRGAI

VFGGGDDPPYGGENPPSEMMELQMIAIHVFNRLYAEIWKSGVALTEREIQCLSWTAAGKT

SAEIAEILGLSEHTVNHYLNQVTRKLDAVNRTQAVVKAIRRGLI

>node293

QARSAEELFELLAKFARNFGFDHFAYGAVDLPSSKPSSLVLSGNYPDEWVERYLEQGYYK

IDPVVRHCRRSTSPFRWSDVQSQQKLEKQQRRVMEEAREFGLRNGLTVPLHGPNGRFAAV

SFAASSHAPYSAEAPPDAMAHLQLLAIQFHERVYARLSSQGVKLTPRERECLHWVAEGKT

SWEIGQILGISENTVNFHLKNVMRKLGATNRTQAVAKAIRLGLI

>node294

AARSAEALFDLMAKAARQFGFDHFAYAHVDLPSSKPSSLVLSGNYPDEWVERYLERNYYL

IDPIHRHCHRTSSPFRWSDVQSLMPLTKQQRRIMEEAREAGLGDGLTVPLHGPGERTASV

SFAARSHAPYSLEAPPDQLLEAQLLASVAHERVYRRLQQRGVKLTPRERECLQWVARGKT

SWEIAQILGISQHTVNEHLKNAMRKLGVTNRTQAVLRALRLGLI

>node295

AARSTKALWDLMRNAMHDYGVKMKAYSHSDVPASKPSGLVAADGFPEEWVCEYLERKLFL

IDPIPQLASRMSRPFRWSEVQDLMPLSQQGERYMETLRDSGLGDGLAMQVYGPGMRNAYV

GLAARSHAPYGFGEPPDQIFELQCVAQVAHLRVYCELRQRGDTLSPREKEVLQWIARGKS

NSVIAEILGISRHTVDAHVRSIFRKLDVTDRTSAALRGLGSGLV

>node296

AARSAEALFDLMAKAARQFGFDHFALAHVDLKSGEPSSLLITGNYPDAWVERYLERNYYL

TDPIHRACHRTSSGFRWSDVQSLIPLTRQQRRILEEAREAGLGDGFTVPSHIPGEPTASV

SFAARSHAPYSLEAPPDQLLLAQLLGSVAYERAYRRLPQRGVRLTPRQRECIQWVARGKT

SWEIAQILGISQETVNEHLKDARRRLGVTNRTQLVLRALRLGLI

>node297

AARSAEALFDLMAKAARQFGFDHFALAHVDLKSGEPSSLLITGNYPDAWVERYLERNYYL

TDPIHRACHRTSSGFRWSDVQSLIPLTRQQRRILEEAREAGLGDGFTVPSHIPGEPTASV

SFAARSHAPYSLEAPPDQLLLAQLLGSVAYERAYRRLPQRGVRLTPRQRECIQWVARGKT

DWEIAQILGISQETVNEHLKDARRRYGVTKRTQLVLRALRDGLI

>node298

AARSAEALFDLMAKAARQMGFDHFALAHVDLKAGEPSSLLIHGNYPDAWVERYLARNLYA

TDPIHRACHRTSSGFRWSDVQSLIPLTRGDRRILEEAREAGLGDGFTVPSHIPGEPTGSC

SFAARSHAPYSLEAPPDQLLLAQLLGSVAFERAYRRLPQRGPRLTERQRECVQWVARGKT

DWEISQILGISQETVIQHLKDARERYGVTKRTLLVLRALFDGLI

>node299

AASSAEQLFEALTKAARQMGFDHFALAHVGRGAGEPSSLLIHGNYPDAWAEVYIGFNLSA

TDPIRRASERSLTGFRWRNVQSLIPLTRGDRQILETARESGLGDGFTVPRHLPGEATGSC

SFAARPHAPYDIPAPPDMLLVAELLGAIAIASAYRQLPQRGPRLTERQRECVLWAARGKT

DWEISQILGISQETVIQHLKDARERYGVHKRSMLILCALFDGLI

>node300

AVRSAEALSDLMAKIARQMGFDHFALAHVDLKAAEPSVLHIHGNYPDAWVERYLARNLYA

TDPIHRACHRTSSGFRWSDVQSLIPLTRGDRRILAEAREAGLGDGFTVPSHIPGEPTGSC

SFAARSHAPYSLEAPPDQLLLAQLLGSFAFERAYRRLPQRGPRLTERQRECVQWVARGKT

DWEISQILGISQETVIQHLKDARERYGVTKRTLLVLRALFDGLI

>node301

QARSAEELLELFAQAALQLGYQHFALCATDLSSSKPPIGVLSHNYPSEWVEHYIERNYYL

IDPVHRHAYRASSPFRWNDVSSQGKREKHQRRIMEEAEDAGLANGLSVPLHEPGGRILLV

SLAASSHAPYSAEAPPDAWAKAYLLAIQFHMRFYRRLQIPGVKLTSRERECLTWVARGKS

SWEIAQILGISEHTVNFHLKNAMAKLNTTSRTLAAVKATRLGLI
